# Biocatalytic Production of Aldehydes: Exploring the Potential of *Lathyrus cicera* Amine Oxidase

**DOI:** 10.3390/biom11101540

**Published:** 2021-10-18

**Authors:** Elisa Di Fabio, Alessio Incocciati, Alberto Boffi, Alessandra Bonamore, Alberto Macone

**Affiliations:** 1 Department of Biochemical Sciences “Alessandro Rossi Fanelli”, Sapienza University of Rome, Piazzale Aldo Moro 5, 00185 Rome, Italy; elisa.difabio@uniroma1.it (E.D.F.); incocciati.1750499@studenti.uniroma1.it (A.I.); alberto.boffi@uniroma1.it (A.B.); 2 Center for Life Nano Science@Sapienza, Istituto Italiano Di Tecnologia, V.le Regina Elena 291, 00161 Rome, Italy

**Keywords:** aldehydes, primary amines, copper-containing amine oxidase, crossflow ultrafiltration, biocatalysis, oxidative deamination, *Lathyrus cicera*

## Abstract

Aldehydes are a class of carbonyl compounds widely used as intermediates in the pharmaceutical, cosmetic and food industries. To date, there are few fully enzymatic methods for synthesizing these highly reactive chemicals. In the present work, we explore the biocatalytic potential of an amino oxidase extracted from the etiolated shoots of *Lathyrus cicera* for the synthesis of value-added aldehydes, starting from the corresponding primary amines. In this frame, we have developed a completely chromatography-free purification protocol based on crossflow ultrafiltration, which makes the production of this enzyme easily scalable. Furthermore, we determined the kinetic parameters of the amine oxidase toward 20 differently substituted aliphatic and aromatic primary amines, and we developed a biocatalytic process for their conversion into the corresponding aldehydes. The reaction occurs in aqueous media at neutral pH in the presence of catalase, which removes the hydrogen peroxide produced during the reaction itself, contributing to the recycling of oxygen. A high conversion (>95%) was achieved within 3 h for all the tested compounds.

## 1. Introduction

Aldehydes are extremely interesting chemical compounds due to their numerous industrial applications. The high reactivity of the carbonyl group of aldehydes makes them versatile feedstocks for the synthesis of resins, dyes, pharmaceutical intermediates through carboligation, condensation, hydrocyanation, transamination, and α-alkylation [1,2,3,4,5]. They can also be used as flavors and fragrances in the cosmetic and food industry [6,7,8]. Many aldehydes, especially those used by the perfume industry, are extracted from natural sources. However, this procedure is typically limited by their intrinsic lability and low bioaccumulation. Given the high-value applications and large markets for several aldehydes and considering the high demand for “natural” labeled compounds, research on their green and sustainable synthesis is taking center stage [9,10,11]. In this frame, biocatalysis occupies a privileged place, being a valid alternative compared to the conventional physical (extraction) or chemical synthetic routes. To date, a variety of engineered microorganisms and isolated enzymes for the de novo biosynthesis of aldehydes have been described and characterized [12,13]. Although microbial aldehyde biosynthetic pathways are well known, their production is typically hindered by their endogenous reduction to the corresponding alcohols [14]. Even if it is possible to limit the production of these unwanted byproducts through microbial engineering (reduced aromatic aldehyde reduction or RARE strain) [15], the resulting accumulation of aldehydes is generally toxic, as they covalently modify proteins, nucleic acids, and coenzymes, affecting the survival of the microorganism itself [16].

A valid alternative to microbial factories is represented by isolated enzymes, both extracted from natural sources and obtained by recombinant protein expression technology [17,18,19].

Aldehydes can be enzymatically synthesized by oxidation of the corresponding primary alcohols [20] and primary amines [21] or by reduction of the corresponding carboxylic acids [22,23,24]. Aldehyde synthesis starting from the corresponding alcohols can be performed by alcohol oxidases, which are typically active on a broad range of alcohols, such as primary and secondary alcohols, allylic and aryl alcohols, sterols, and carbohydrates [25,26]. However, the major drawback of these enzymes is the overoxidation of the aldehydes to carboxylic acids. In addition, they are not active on functionalized β-ethyl alcohols [27]. Conversely, alcohol dehydrogenases require expensive cofactors and are active on a limited selection of substrates [28]. Aldehydes can be also obtained starting from the corresponding carboxylic acids through the action of broad substrate specificity carboxylic acid reductases [29,30]. However, enzymatic reduction of the carboxylic acids to the corresponding aldehydes is an energetically demanding reaction that requires the use of ATP as a co-substrate. In addition, this reaction is hard to control due to the further reduction of aldehydes to alcohols. Thus, in industrial syntheses, carboxylic acid reductases are typically coupled to other enzymes in domino processes to quickly carry out decarbonylation, reduction, or transamination of the newly synthesized aldehydes. A further way for the enzymatic synthesis of aldehydes is the oxidative deamination of the corresponding amines. From a biocatalysis point of view, this is still a rather unexplored field. To date, the enzymes capable of catalyzing this reaction, amine oxidases, have been mainly used in the development of biosensors [31,32]. More recently, plant diamine oxidases (DAO) are emerging as attractive biocatalysts for the one-step bioconversion of primary amines to the corresponding aldehydes. These enzymes, belonging to the family of copper-containing amine oxidases, catalyze the oxidative deamination of polyamines such as putrescine, cadaverine and spermidine, with the concomitant production of H_2_O_2_. The production of hydrogen peroxide is physiologically involved in cell wall maturation during plant development as well as in defense mechanisms during pathogen invasion [33,34]. Unlike mammalian and prokaryotic DAOs, plant enzymes display higher chemical stability and turnover rate, making them attractive for biotransformations. To date, plant DAOs from various species have been purified to homogeneity and characterized, the best known and studied being those from *Leguminosae sp* [35,36]. However, until now, the use of these DAOs as biocatalysts has been little explored. Our research group used *Lathyrus cicera* diamine oxidase (LCAO) in a domino process for the synthesis of new non-natural benzylisoquinoline alkaloids [19]. In this frame, LCAO showed a relaxed substrate specificity, being active on four different β-substituted ethylamines. However, in this catalytic process, aldehydes did not accumulate as they were transient intermediates in the Pictet–Spengler condensation catalyzed by *T. flavum* norcoclaurine synthase recombinantly expressed in *E. coli* [37].

In the present work, we have explored the biocatalytic potential of LCAO by developing a general method for the synthesis of pure aldehydes starting from differently substituted aliphatic and aromatic primary amines. The synthesis of these aldehydes is particularly interesting for several reasons: (i) they are not commercially available; (ii) some of them are biogenic aldehydes that can be used as pure standards for measurements in human and animal tissues; (iii) they can be used in domino reactions as substrates of other enzymes to produce intermediates of active pharmaceutical ingredients [38]. In addition, to make the whole process fast, easy, and scalable, we developed a new chromatography-free purification protocol of LCAO extracted from *Lathyrus cicera* seedlings.

## 2. Materials and Methods

### 2.1. Chemicals

Reagents and solvents obtained from commercial suppliers were used without further purification. All chemicals were purchased from Sigma-Aldrich Srl, Milan, Italy.

### 2.2. LCAO Chromatography-Free Purification

*Lathyrus cicera* seeds (300 g) were soaked for 12 h in tap water and then spread in a monolayer on trays lined with moistened filter paper. The trays were covered tightly with aluminum foils. The germinated seeds were watered every 2 days with 100 mL of tap water. After 10 days, the etiolated seedlings (about 500 g) were harvested above the roots, cut in small pieces (about 2 mm) and washed with 1 L of ice-cold deionized water for 15 min. After washing, the ground plant sample was kept in 1.5 L of ice-cold extraction buffer (50 mM pH 5.5 phosphate buffer containing 0.3 M NaCl) for 30 min and then filtered through a 20 Micron Nylon Mesh and squeezed by hand to remove the debris. To allow quantitative recovery, the pellet was extracted again as described above and the resulting crude mixtures were combined. To remove insoluble material, the crude enzyme extract (3 L) was clarified by disposable Sartolab^®^ Vacuum Filters System (Polyethersulfone, 0.22 μm) using 30 g of Sartoclear Dynamics^®^ Lab Filter Aid (Sartorius) containing highly pure diatomaceous earth (Celpure^®^ C300–pharmaceutical grade) pre-wetted with ultrapure water. The buffer was exchanged with 20 mM phosphate buffer pH 6.5 through diafiltration by means of crossflow ultrafiltration using a single Vivaflow 200 module (Sartorius) with a 50 kDa cutoff, coupled to a Masterflex L/S pump system. The same device was also used to concentrate the enzyme extract to 100 mL. The feed flow rate was set to 40 mL/min both in concentration and diafiltration modes. As the last purification step, the sample was subjected to heat treatment at 65 °C for 15 min. Denatured proteins were removed by centrifugation (12,000 rpm, 20 min, 20 °C). All purification steps were monitored by SDS-PAGE (12% SDS) and measuring the enzyme’s specific activity. Enzyme activity (LCAO Units) was used to determine the recovery after each purification step. Total soluble protein concentration was determined using the method of Bradford with bovine serum albumin as a standard. LCAO content throughout the purification procedure was also monitored by high-performance size exclusion chromatography (HP-SEC). HP-SEC was performed using an Agilent Infinity 1260 HPLC apparatus equipped with a UV detector. Separation was carried out using an Agilent AdvanceBio SEC 300 Å, 7.8 × 150 mm, 2.7 µm, LC column connected to an AdvanceBio SEC 300 Å, 7.8 × 50 mm, 2.7 µm, LC guard column. Isocratic analysis was carried out with 20 mM phosphate buffer pH 6.5 containing 150 mM NaCl as mobile phase. The flow rate was 0.7 mL/min over an elution window of 7 min. Protein elution was followed using UV detection at 220 nm and 445 nm (TPQ-phenylhydrazine adduct).

### 2.3. LCAO Acitivity

LCAO activity was assayed by a coupled diamine oxidase/peroxidase spectrophotometric test at 25 °C with 10 mM putrescine in 20 mM phosphate buffer at pH 6, 7, and 8 in the presence of 150 nM enzyme. H_2_O_2_ produced by LCAO was monitored following horse radish peroxidase catalyzed coupling of 4-aminoantipyrine (AAP) and sodium 3,5-dichloro-2 hydroxybenzenesulfonate (DCHBS) as oxygen acceptor. The enzymatic activity, which led to the formation of colored product, was measured at 515 nm for 60 s and expressed as U/mL per mg protein. Initial rates of reaction at the various substrate concentrations were determined at three different pH values (6, 7, 8), and the kinetic parameters were calculated by non-linear regression fitting of the data to the Michaelis–Menten equation V = Vmax(S)/(Km + (S)). All curve fitting was carried out using Kaleidagraph software (Synergy Software, Reading, PA, USA).

### 2.4. Enzymatic Synthesis of Aldehydes

The biocatalytic synthesis of the aldehydes was carried out starting from the corresponding primary amines in the presence of LCAO. Twenty different substituted ethyl amines were tested. A solution of 5 mM of amine substrate was prepared in 50 mM HEPES buffer pH 7.0 in the presence of 250 U/mL of catalase from bovine liver (Sigma-Aldrich) to a final volume of 10 mL. LCAO was added to a final concentration of 2 U/mL and the reaction was carried out at room temperature under stirring. To ensure constant catalytic efficiency, the enzyme is supplied (5 U) at regular time intervals (30 min). The substrate consumption was monitored by HPLC or GC/MS, whereas aldehyde formation was monitored by purpald^®^ assay.

Aromatic amine consumption was followed by HPLC using an Agilent Infinity 1260 HPLC apparatus equipped with UV and fluorometric detectors. The separation was carried out using a Halo C18 AQ column (3 × 150 mm, 2.7 µm) connected to the C18 AQ guard column (3 × 5 mm, 2.7 µm). The elution was performed at a flow rate of 0.8 mL/min, with solvent A (0.1% trifluoroacetic acid in water) and solvent B (0.1% trifluoroacetic acid in acetonitrile). The mobile phase was linearly increased from 0% to 100% of solvent B in 15 min and then run isocratically for 5 min. Afterward, buffer A was reintroduced in the mobile phase up to 100%, and the column was allowed to equilibrate for 10 min. The elution profile of aromatic amines was monitored by setting the UV detector at 280 nm.

Aliphatic amine consumption was followed by GC/MS. Derivatization with ethyl chloroformate (ECF) was conducted in a single step by adding to 0.1 mL of the reaction mixture 20 μL of ECF dissolved in 0.5 mL of dichloromethane. The biphasic system was stirred vigorously for 2 min, saturated with NaCl and extracted sequentially with 1 mL of ethyl ether and 1 mL of ethyl acetate. The organic extracts were combined, dried under nitrogen flow, resuspended in dichloromethane, and analyzed by GC/MS. GC/MS analyses were performed with an Agilent 6850A gas chromatograph coupled to a 5973N quadrupole mass selective detector (Agilent Technologies, Palo Alto, CA, USA). Chromatographic separations were carried out with an Agilent HP-5ms fused silica capillary column (30 m × 0.25 mm id) coated with 5% phenyl–95% dimethylpolysiloxane (film thickness 0.25 μm) as stationary phase. Injection mode: splitless at a temperature of 280 °C. Column temperature program: 100 °C for 2 min and then to 300 °C at a rate of 15 °C/min and held for 5 min. The carrier gas was helium at a constant flow of 1.0 mL/min. The spectra were obtained in the electron impact mode at 70 eV ionization energy; ion source 280 °C; ion source vacuum 10-5 Torr. Mass spectrometric analysis was performed in the range m/z 50 to 500 at a rate of 0.42 scans s^−1^).

### 2.5. Purpald^®^ Colorimetric Assay

Substrate conversion efficiency was calculated through the reaction of the newly formed aldehyde with 4-Amino-5-hydrazino-1,2,4-triazole-3-thiol (purpald^®^), a reagent for the colorimetric detection of aldehydes. A volume of 50 μL of the reaction mix were added to 1 mL NaOH 2 M containing 5 mg of purpald^®^, vortexed for 5 min and read at 550 nm after 15 min. The calibration was carried out using phenylacetaldehyde commercial standard in the concentration range 0.28–7 mM.

## 3. Results and Discussion

### 3.1. LCAO Purification

**Figure 1:**
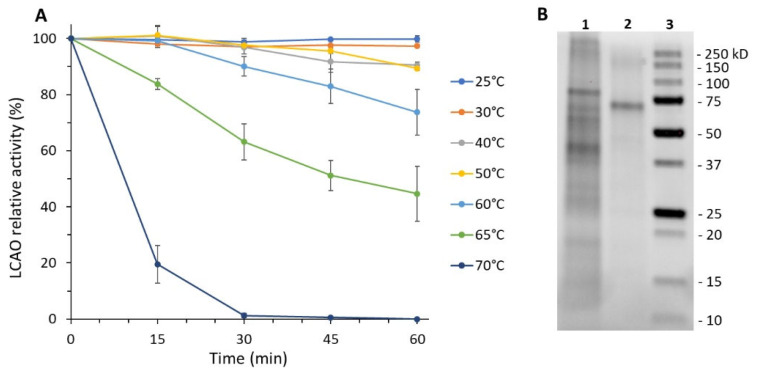
Heat treatment was used as the last step of LCAO purification. (**A**) Thermal stability of LCAO in the range 25–70 °C. (**B**) SDS-PAGE of LCAO after the heat treatment at 65 °C for 15 min. Lane 1: insoluble fraction; lane 2: soluble fraction; lane 3: protein ladder.

In the present paper, we have developed a new protocol for the purification of LCAO, which has significant advantages over the generally used one, as it is chromatography-free, fast, and easily scalable. Sprouts of *L. cicera* were selected because they show higher specific activity than other common Leguminosae, such as Pisum sativum, Lens culinaris, and Phaseolus vulgaris (data not shown). Recently, our research group has developed a protocol that involves the combined use of diatomaceous earth filter aid and tangential ultrafiltration for the purification of recombinant proteins [39]. Now, for the first time, these techniques are being used on plant material for the purification of LCAO. According to this purification strategy, the crude plant extract is vacuum filtered using diatomaceous earth as a filter aid. This step replaces the classic centrifugation steps which are time-consuming and limit the amount of plant material that can be processed. As shown in Table 1, this allows an almost total recovery of the enzymatic activity with a concomitant increase in specific activity. The subsequent diafiltration/ultrafiltration step has a dual objective: (i) exchange the buffer, bringing the pH and ionic strength values to those that guarantee greater stability of the enzyme and, (ii) concentrate the enzyme itself. In addition, in this case, all the enzymatic activity is maintained and there is a 12-fold increase in the specific activity. Tangential ultrafiltration replaces classical dialysis and concentration steps, making the whole process fast and efficient. This process can be easily scaled up using suitable crossflow systems equipped with cassettes with larger membrane areas, able to filter larger volumes (up to thousands of liters). Considering that tangential flow filtration devices and cassettes can be cleaned and reused several times, the system is also economical. Since the stability of the enzyme is not affected by temperatures up to 60 °C (Figure 1A), the last purification step consists of a mild heat treatment, which leads to a 22-fold increase in specific activity, comparable to that obtained by classical chromatographic purification.

As demonstrated by SDS-PAGE and HP-SEC analysis (Figure 1B, Figures S1 and S2) LCAO is highly purified (>95%). The whole purification procedure takes about 7 h compared to the 16 h needed for the previously used protocol. The purified protein is stable for up to 6 months, sterile filtered at 4 °C (Figure S3).

### 3.2. Exploring LCAO Activity toward Aliphatic and Aromatic Primary Amines

**Scheme 1:**
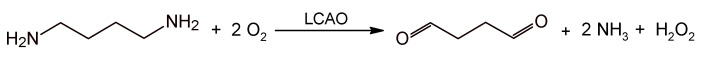
LCAO catalyzed conversion of putrescine into the corresponding aldehyde.

LCAO typically uses as a substrate of choice small polyamines such as putrescine, cadaverine, and spermidine, which are oxidatively deaminated with the production of hydrogen peroxide (Scheme 1).

Similarly, to other copper amine oxidases, it has been reported that this enzyme is also able to accept substrates of a different nature (e.g., histamine, tyramine, benzylamine, etc.) although with lower catalytic performances [36,40]. We, therefore, decided to test the enzymatic activity of LCAO against a larger number of substrates to obtain a variety of aldehydes that may be used as intermediates for the synthesis of molecules with potentially interesting pharmacological profiles. Since this biocatalytic process occurs in aqueous media, aldehydes can be actually used in domino processes by adding other enzymes that use them as substrates [19,40,41] or in other organic transformations that can be carried out directly in water [42,43,44]. In this study, we tested the activity of LCAO toward different classes of commercially available primary amines: 14 substituted β-ethylamines, three substituted γ-propylamines and three linear amines (Table 2 and Figure S6).

LCAO kinetic parameters were determined at pH 6, 7, and 8. The enzyme is inactive at pH 6, while it is active at pH 7 on all the tested substrates with the exception of compound **9a**, which can be considered a poor substrate. This molecule is processed slowly by the enzyme, and it was not possible to accurately measure the kinetic parameters. This could be probably due to the presence of the hydroxyl groups in the catechol moiety. The enzyme is active in the absence of substituents on the aromatic ring (**2a**), in the presence of substituents in the para position (**1a**, **10a**), or when the catechol −OH are singly or both methylated (**3a**–**9a**). Besides phenylethylamines, LCAO can also transform non-aromatic or heterocyclic ethyl- or propylamines (**11a**–**17a**) as well as linear amines (**18a**–**20a**). Conversely, the enzyme is generally less performing at pH 8. At this pH value, compounds **9a**, **15a** and **17a** are not transformed at all. To date, LCAO crystal structure is not yet available, and it is challenging to establish the structural determinants of the interaction between the enzyme and these unnatural substrates. Although the catalytic performance of LCAO toward all the tested amino substrates is lower than that measured for putrescine, the natural substrate (k_cat_ = 262 s^−1^ and K_M_ = 2.7 × 10^−4^ M) [36], the kinetic data indicate that this enzyme can actually be used for synthetic purposes.

### 3.3. Biocatalytic Production of Aldehydes

**Scheme 2:**
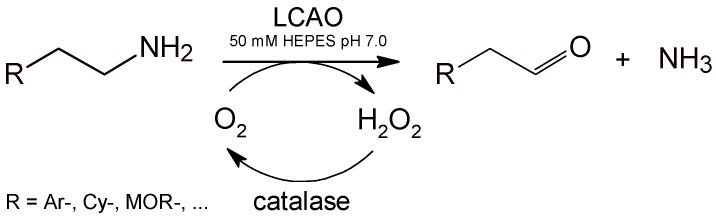
Biocatalytic conversion of primary amines (compounds **1a**–**20a**) into the corresponding aldehydes (compounds **1b**‒**20b**) in the presence of LCAO and catalase.

Considering that LCAO shows the best catalytic performance at pH 7, the synthesis of the aldehydes was carried out at this pH value starting from the corresponding primary amines (Scheme 2).

In the biocatalytic production of aldehydes, LCAO is coupled with catalase, which decomposes hydrogen peroxide, a co-product of the reaction, to water and oxygen. Hydrogen peroxide must be quickly removed from the reaction medium because it can inactivate the proteins and oxidize the substrates and the reaction products. Thus, catalase preserves LCAO activity, while contributing to the recycling of oxygen which is co-substrate of the reaction.

The production of the aldehydes was monitored by the purpald^®^ colorimetric assay that, unlike the time-consuming GC and HPLC methods, is typically fast, while preserving high specificity and sensitivity. The assay was set up using the commercially available phenylacetaldehyde as a standard (Figure S4). The reaction products were also characterized by GC/MS by comparison with the fragmentation profiles of the NIST2017 database (Figure S5).

As shown in Table 3 (and Table S1), all the tested amines are about completely converted into the corresponding aldehydes (**1b**–**20b**) in a time ranging from 0.5 to 3 h. The formation of byproducts, such as imine derivatives (deriving from the reaction between aldehydes and primary amines or ammonia), which typically form at pH 4–5, is not observed at pH 7.

The enzyme is efficient in the conversion of linear amines (compounds **19a** and **20a**), structurally similar to the natural substrate, and with para-substituted phenylethylamines (compounds **1a** and **10a**). For these compounds, the transformation occurs within an hour. To ensure constant catalytic efficiency, the enzyme is supplied at regular time intervals (30 min). This is necessary because, over longer reaction times, the newly synthesized aldehydes may partially inactivate the enzyme. Considering the intrinsic reactivity and instability of aldehydes, this biosynthetic method is particularly suitable for their use as short-living intermediates in domino processes where the aldehydes can be quickly processed in enzymatic cascades [19,41]. However, to evaluate the scalability of the process, we applied the synthesis protocol described above to the conversion of a 10-fold amount of **2a** to the corresponding aldehyde. In addition, in this case, we had an almost total conversion. The extraction of aldehyde from the aqueous reaction media resulted in yield of isolated product of 43 mg (71.6%, purity > 95%) (Figure S7).

## 4. Conclusions

Aldehydes are extremely interesting chemical compounds for their numerous industrial applications. Considering the high demand for “naturally” synthesized aldehydes, especially in food and cosmetic industries as well as their use for pharmaceutical application, the development of a new green enzymatic protocol for their production is of great interest. In this study, we set up a fully enzymatic strategy for the synthesis of aldehydes by means of a plant extracted amine oxidase. We have shown that this enzyme has a broader substrate specificity than that known thus far, and it is able to catalyze the oxidative deamination of all the amines tested except for dopamine. Structural and site-specific mutagenesis studies will help to gain a deeper understanding of the structural determinants need for recognition of the substrate and its subsequent transformation. Despite an excellent substrate conversion (>95%), the synthesis of aldehydes is still limited to low milligram scale. However, the immobilization and/or modification of the enzyme surface to minimize aldehyde-enzyme interactions could make the scale-up of the process feasible. In addition, the chromatography-free LCAO purification protocol developed here will help make the overall synthetic process easy and cost-effective.

## Figures and Tables

**Table 1:** Purification procedure for *Lathyrus cicera* amine oxidase. The recovery (%) was determined by evaluating the total activity (enzyme units) after each purification step.

Purification Step	Total Activity (Units)	Total Protein (mg)	Specific Activity (units × mg^−1^ Protein)	Recovery (%)	Purification (-fold)
Crude extract	2630	2706	0.97	100	1
Filtration on Celpure^®^ C300	2590	1546	1.68	98.47	1.73
Vivaflow200 diafiltration/ultrafiltration	2606	217	12.01	99.08	12.38
Heat treatment (65 °C)	2100	97.3	21.58	79.85	22.24

**Table 2:** Steady-state kinetic parameters for LCAO-catalyzed oxidative deamination of primary amines.

			pH 7				pH 8		
n.	Structure	K_M_ (mM)	k_cat_ (s^−1^)	k_cat_/K_M_ (M^−1^s^−1^)	Relative Activity (%)	K_M_ (mM)	k_cat_ (s^−1^)	k_cat_/K_M_ (M^−1^s^−1^)	Relative Activity (%)
**1a**	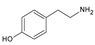	0.60	29.24	49.00	100	0.49	13.30	26.94	100
**2a**	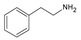	1.20	18.35	14.25	62.8	0.60	9.16	15.92	68.9
**3a**	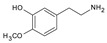	1.58	0.27	0.21	0.9	1.66	0.17	0.10	1.3
**4a**	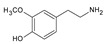	1.31	2.00	1.53	6.8	1.35	0.96	0.69	7.2
**5a**	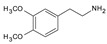	1.00	1.54	1.53	5.3	1.80	2.31	1.26	17.4
**6a**	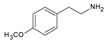	5.37	2.53	0.45	8.7	5.60	4.02	0.70	30.2
**7a**	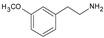	0.47	6.20	13.04	21.2	1.20	1.80	1.48	13.5
**8a**	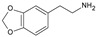	3.83	2.95	0.77	10.1	1.44	1.21	0.78	9.1
**9a**	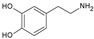	ND	ND	0.49	ND	ND	ND	ND	ND
**10a**	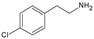	3.38	10.15	3.15	34.7	1.30	1.98	1.45	14.9
**11a**	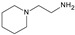	0.42	5.53	0.79	18.9	0.70	0.73	0.23	5.5
**12a**	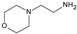	6.46	5.63	0.72	19.3	0.75	0.17	0.41	1.3
**13a**	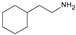	1.44	1.06	0.79	3.6	0.70	0.29	0.80	2.2
**14a**	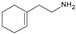	1.90	1.47	12.93	5.0	0.23	0.19	1.04	1.4
**15a**	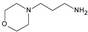	17.00	1.27	0.07	4.3	ND	ND	0.00	ND
**16a**	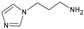	0.61	2.77	4.92	9.5	0.25	0.40	1.60	3.0
**17a**	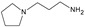	2.30	5.08	2.14	17.4	ND	ND	0.46	0.0
**18a**	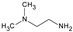	0.23	1.27	2.05	4.3	0.11	2.20	2.19	16.5
**19a**	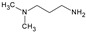	8.80	17.9	1.29	61.2	0.33	0.73	5.47	5.5
**20a**	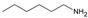	2.59	3.51	5.35	12.0	2.12	11.8	20.00	88.7

**Table 3:** Conversion of the primary amines **1a**–**8a**, **10a**–**20a** to the corresponding aldehydes **1b**–**8b**, **10b**–**20b**.

n.	Reaction Product	LCAO(Total Units)	Conversion (%)			
			0.5 h	1 h	2 h	3 h
**1b**	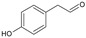	25		99		
**2b**	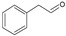	25		98		
**3b**	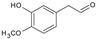	45				95
**4b**	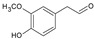	45				97
**5b**	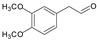	45				96
**6b**	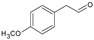	45				98
**7b**	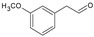	45				99
**8b**	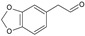	45				95
**10b**	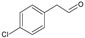	20	98			
**11b**	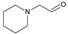	25		98		
**12b**	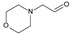	35			99	
**13b**	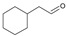	45				98
**14b**	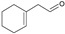	45				97
**15b**	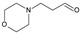	35			96	
**16b**	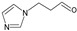	35			96	
**17b**	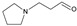	35			97	
**18b**	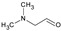	45				98
**19b**	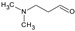	20	99			
**20b**	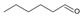	25		99		

0, 10, 20, 30, 40, 50, 60, 70, 80, 90, 100.

## Data Availability

All data related to the manuscript are available in the manuscript and in the Supplementary Information in the form graphs, figures, and tables.

## Supplementary Materials

The following are available online at https://www.mdpi.com/article/10.3390/biom11101540/s1, Figure S1: HP-SEC analysis of purified LCAO, Figure S2: UV spectrum of purified LCAO with or without phenylhydrazine, Figure S3: Overtime stability of sterile-filtered LCAO, stored at 4 °C, Figure S4: Purpald^®^ reaction scheme and calibration plot using standard phenylacetaldehyde, Figure S5: electron impact (70 eV) mass spectra of compounds **1b**–**8b**, **10b**–**20b**, Figure S6: Kinetic plots of LCAO catalyzed deamination of compounds **1a**–**8a**, **10a**–**20a**, Figure S7: enzymatic synthesis of compound **2b**: HPLC and GC/MS analyses, Table S1: Conversion (%) of the amines **1a**–**8a**, **10a**–**20a** to the corresponding aldehydes **1b**–**8b**, **10b**–**20b**. Table S2: HPLC and GC/MS retention times of compounds **1a**–**8a**, **10a**–**20a**, **1b**–**8b**, **10b**–**20b**.
