# Supplementary material for: Biocatalytic Production of Aldehydes: Exploring the Potential of Lathyrus cicera Amine Oxidase
Source: Biomolecules. 2021 Oct 18;11(10):1540. doi: 10.3390/biom11101540 (PMC8533949; doi:10.3390/biom11101540)
Supplement: Supplementary file 1 [file biomolecules-11-01540-s001.zip › biomolecules-1390721-supplementary.pdf]

SUPPORTING INFORMATION

Biocatalytic production of aldehydes: exploring the potential of *Lathyrus cicera* amine oxidase

Elisa Di Fabio, Alessio Incocciati, Alberto Boffi,
Alessandra Bonamore, Alberto Macone

Figure S1: HP-SEC analysis of purified LCAO

Figure S2: UV spectrum of purified LCAO with or without phenylhydrazine

Figure S3: Overtime stability of sterile-filtered LCAO, stored at 4°C

Figure S4: Purpald® reaction scheme and calibration plot using standard phenylacetaldehyde

Figure S5: Electron impact (70eV) mass spectra of compounds **1b**-**8b**, **10b**-**20b**

Figure S6: Kinetic plots of LCAO catalyzed deamination of compounds **1a**-**8a**, **10a**-**20a**

Figure S7: Enzymatic synthesis of phenylacetaldehyde (compound **2b**): HPLC and GC/MS analyses

Table S1: Conversion (%) of the amines **1a-8a**, **10a-20a** to the corresponding aldehydes **1b-8b, 10b-20b.**

Table S2: HPLC and GC/MS retention times of compounds **1a-8a**, **10a-20a, 1b-8b, 10b-20b.**

Figure S1: **HP-SEC analysis of purified LCAO**.

Highly purified LCAO was analyzed by HP-SEC. A) chromatographic profile following the UV signal at 220 nm; B) chromatographic profile following the UV signal of TPQ-phenylhydrazine adduct (445 nm). Phenylhydrazine typically forms a covalent adduct with TPQ that can be followed spectroscopically at 445 nm. The signal of the TPQ-phenylhydrazine adduct at the retention time of the LCAO allows to unequivocally identify this enzyme.

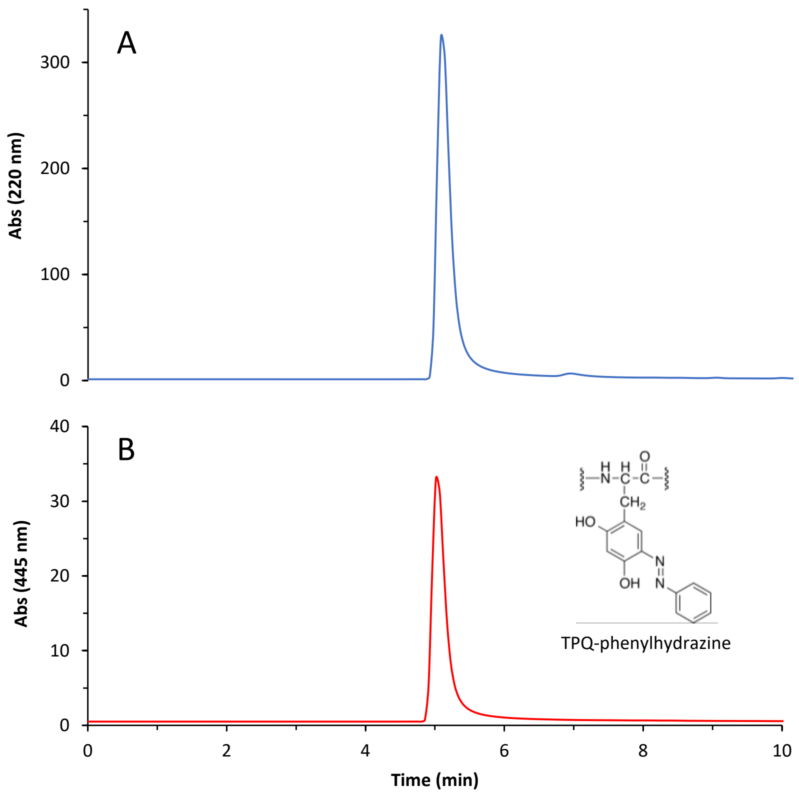

Figure S2: **UV-vis spectrum of purified LCAO**

UV-vis spectra were acquired with (red line) or without (blue line) phenylhydrazine that covalently bounds TPQ cofactor at the active site of LCAO. the TPQ- phenylhydrazine adduct formation leads to a spectrum change in the visible region (400-500 nm).

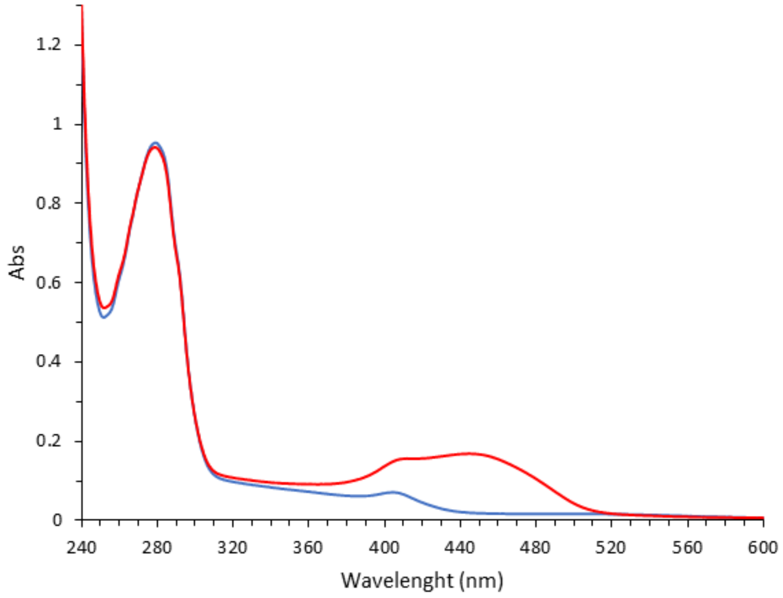

Figure S3: Overtime stability of LCAO

Higly purified LCAO was sterile filtered and stored at 4°C. Enzyme activity was assayed regularly over 6 months. During this time frame, the enzymatic activity does not change.

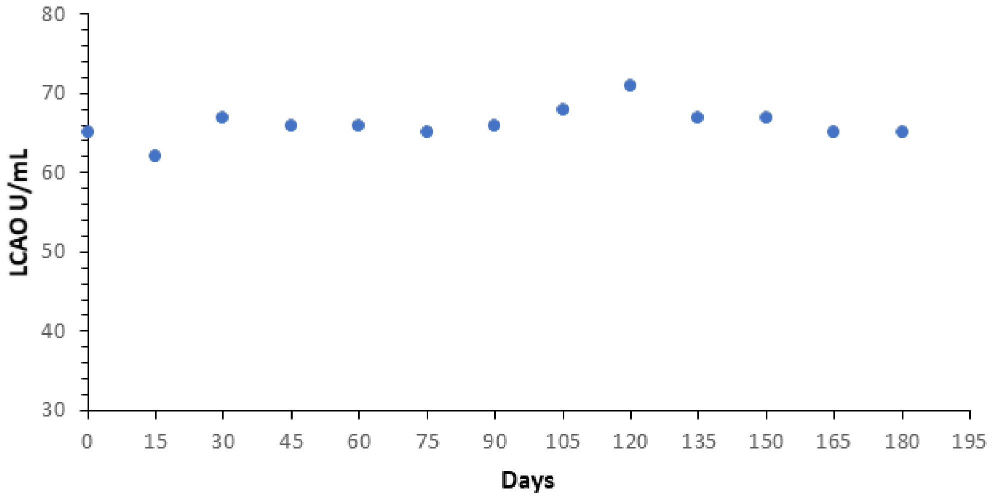

Figure S4: **Purpald® calibration plot**

Purpald® calibration plot was performed using commercial phenylacetaldehyde (0.28-7.0 mM) as standard (left panel); Purpald® reaction with aldehydes leading to the formation of a colored product (550nm) (right panel).

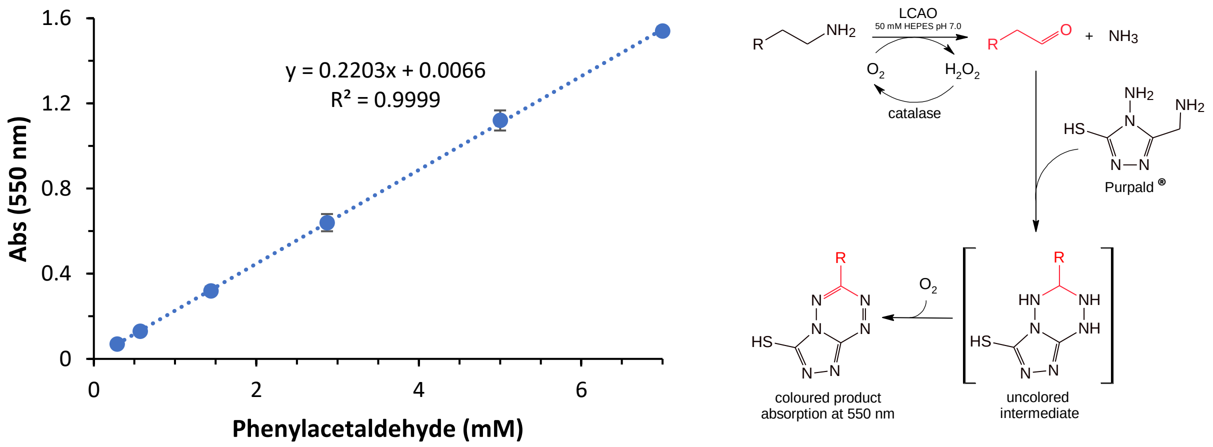

Figure S5: Electron impact (70eV) mass spectra of compounds 1b-8b, 10b-20b

All the enzymatically synthesized aldehydes were extracted from the reaction mixture and characterized by GC-MS. All the fragmentation profiles obtained by electronic impact (70 eV) are reported below.

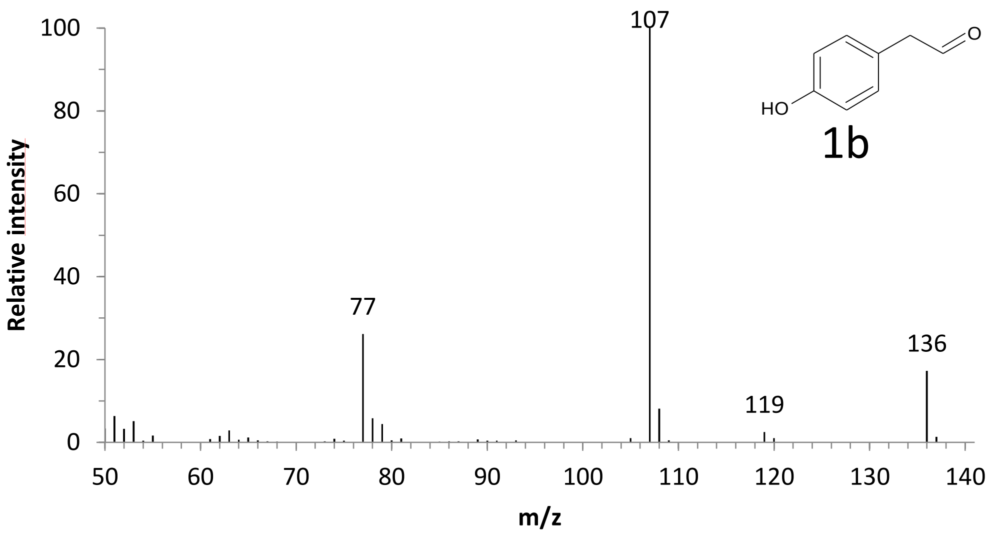

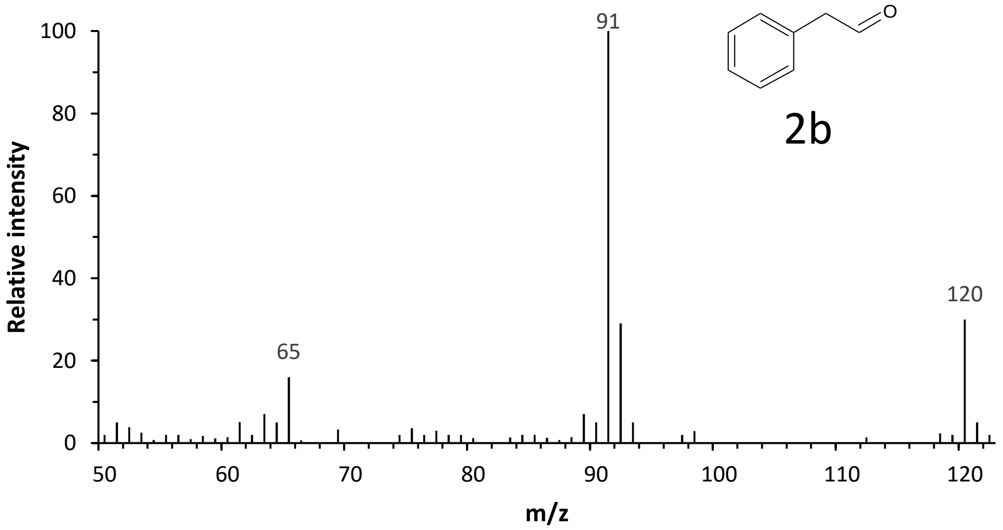

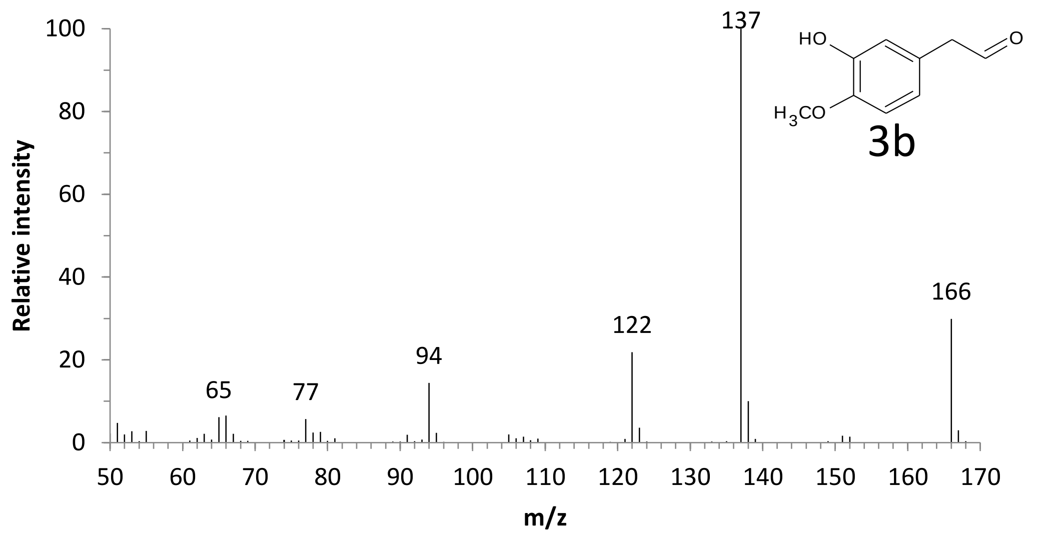

137
HO
O
H3CO
3b
100
80
60
40
20
0
Relative intensity
65
77
94
122
166
50
60
70
80
90
100
110
120
130
140
150
160
170
m/z

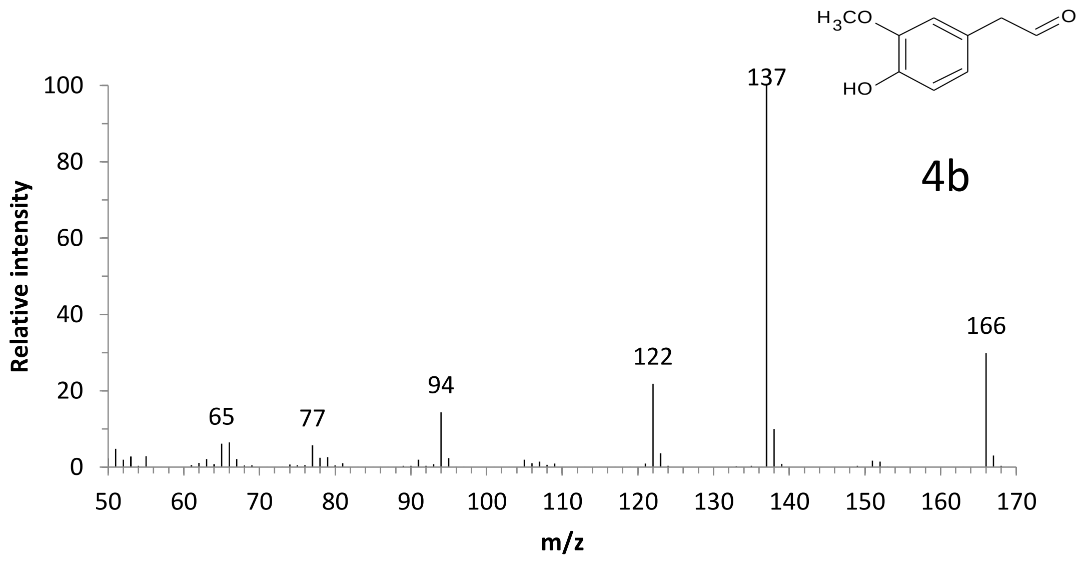

H3CO
O
HO
137
4b
100
80
60
40
20
0
Relative intensity
65
77
94
122
166
50
60
70
80
90
100
110
120
130
140
150
160
170
m/z

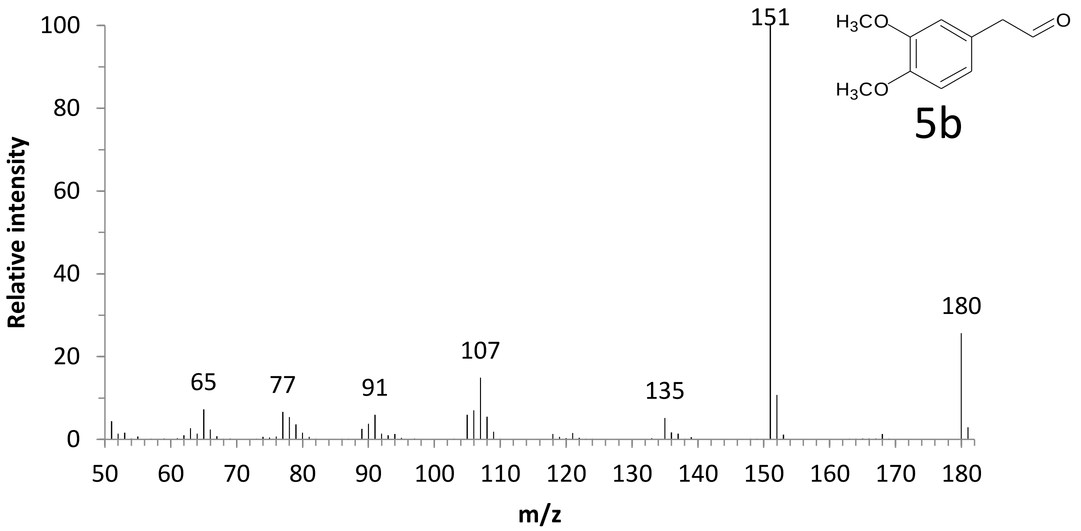

Relative intensity
m/z
151
180
107
135
65
77
91
H3CO
H3CO
O

5b

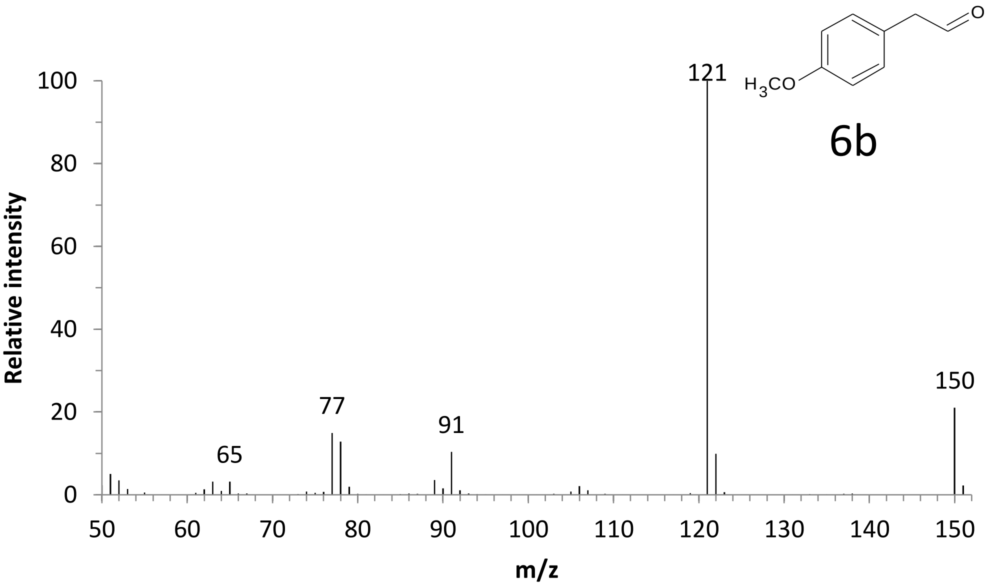

Relative intensity
m/z
121
150
77
91
65
H3CO
O

6b

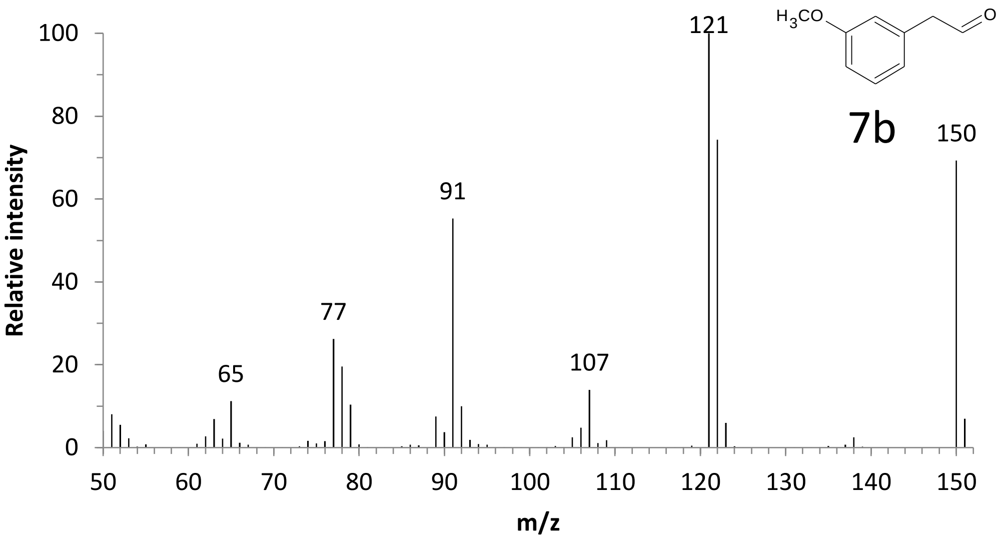

H3CO
O
7b
121
150
91
77
65
107
Relative intensity
m/z
100
80
60
40
20
0
50
60
70
80
90
100
110
120
130
140
150

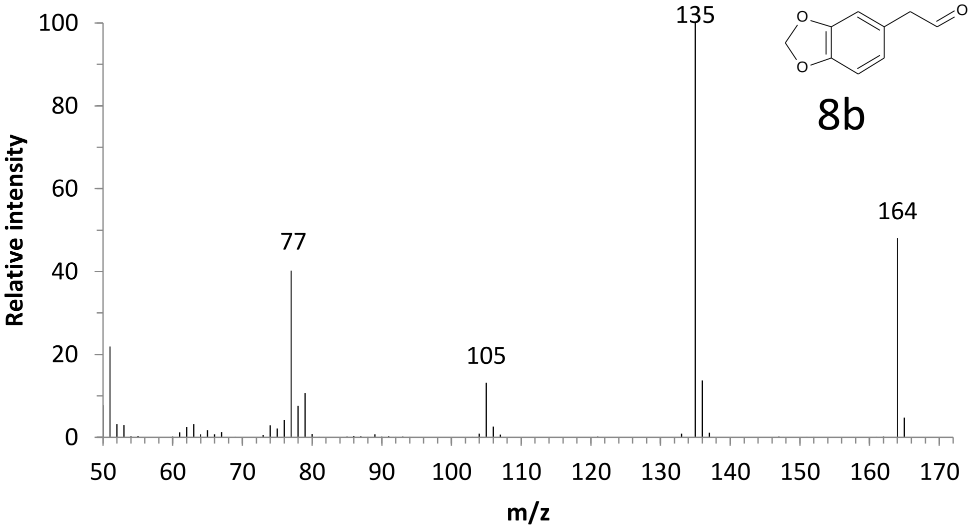

O
O
O
8b
135
164
77
105
Relative intensity
m/z
100
80
60
40
20
0
50
60
70
80
90
100
110
120
130
140
150
160
170

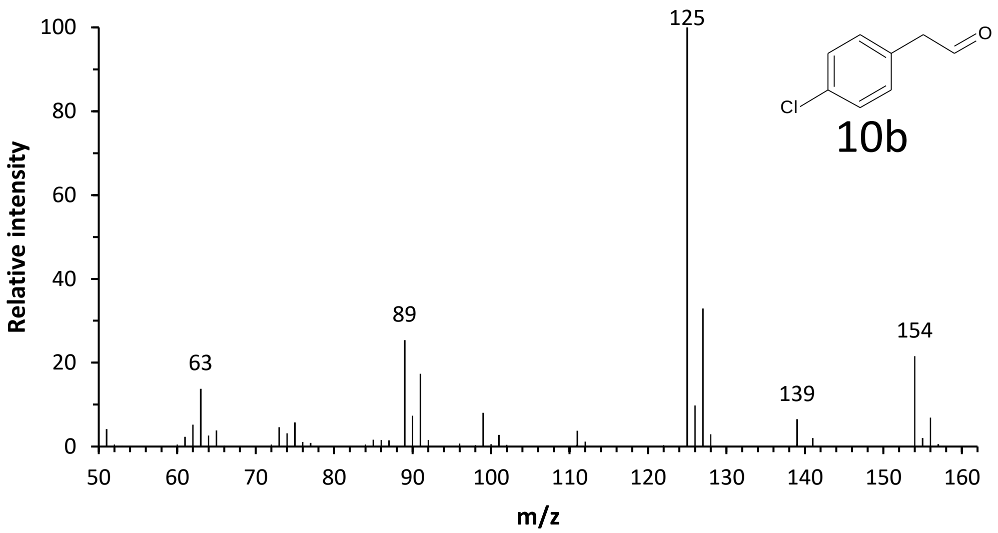

125
Cl
O
10b
89
63
154
139
Relative intensity
m/z

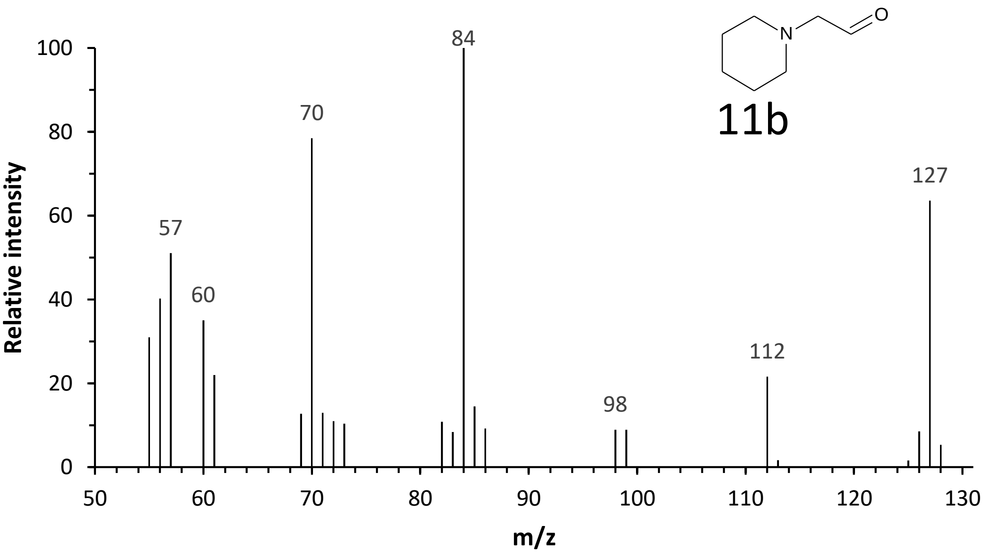

N
O
11b
84
70
57
60
127
112
98
Relative intensity
m/z

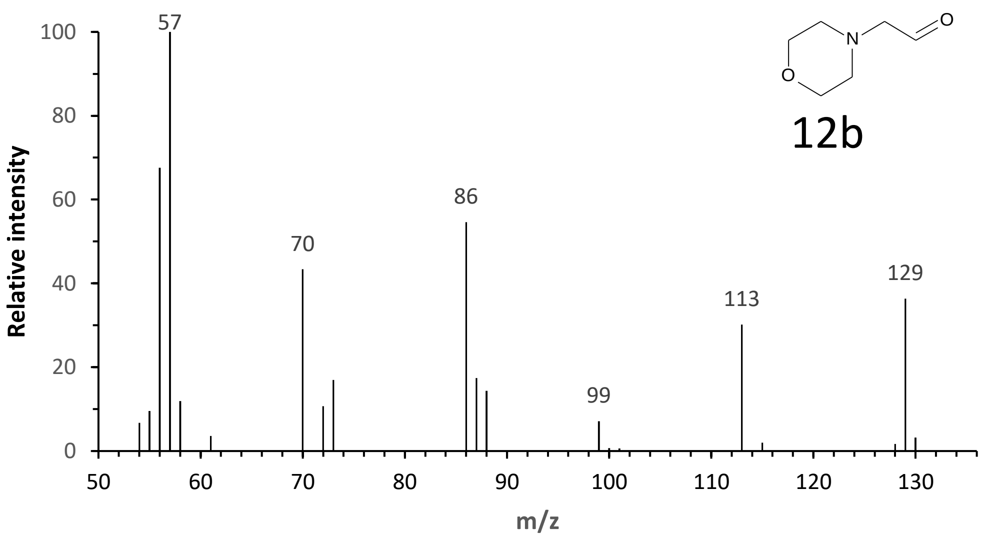

100
80
60
40
20
0
Relative intensity
57
70
86
99
113
129
50
60
70
80
90
100
110
120
130
m/z
N
O
O
12b

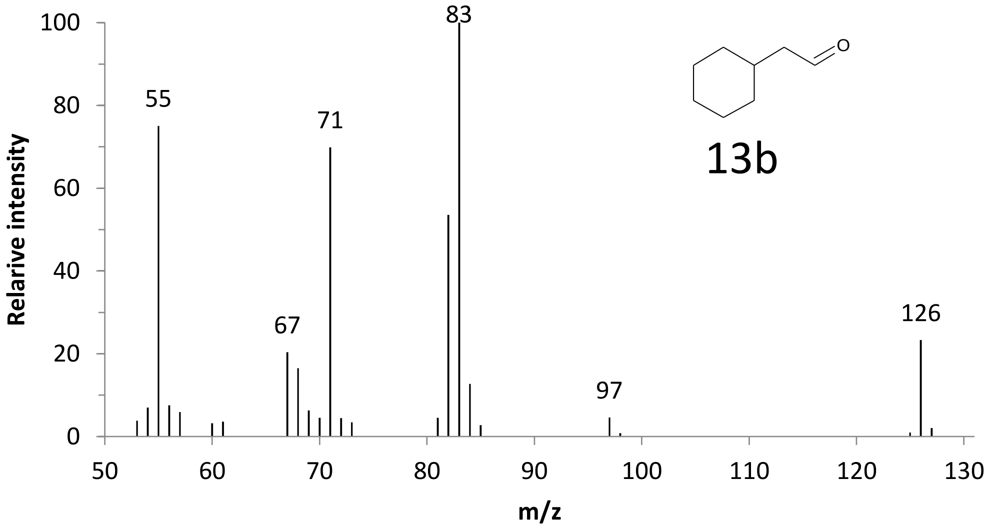

100
80
60
40
20
0
Relarive intensity
55
67
71
83
97
126
50
60
70
80
90
100
110
120
130
m/z
O
13b

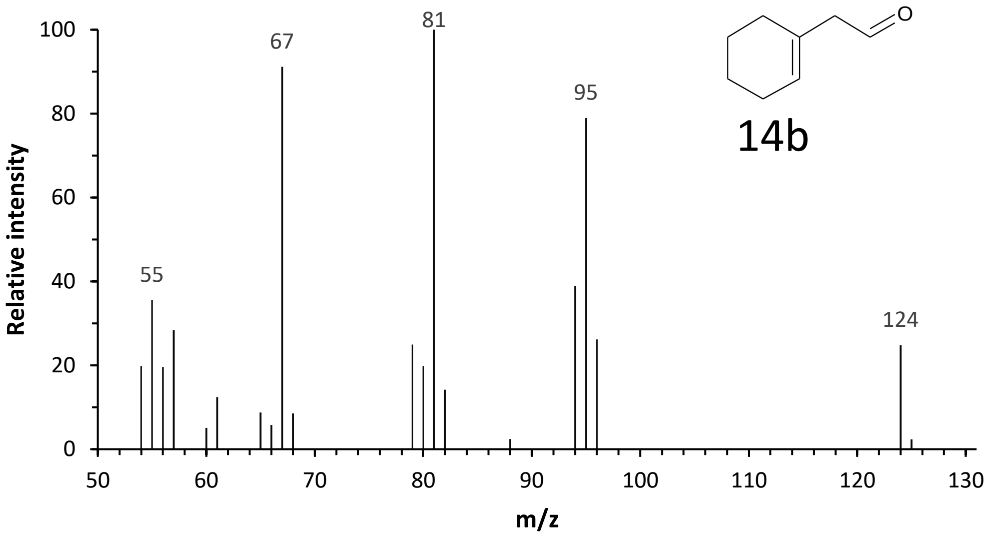

Relative intensity
m/z
55
67
81
95
124
O
14b

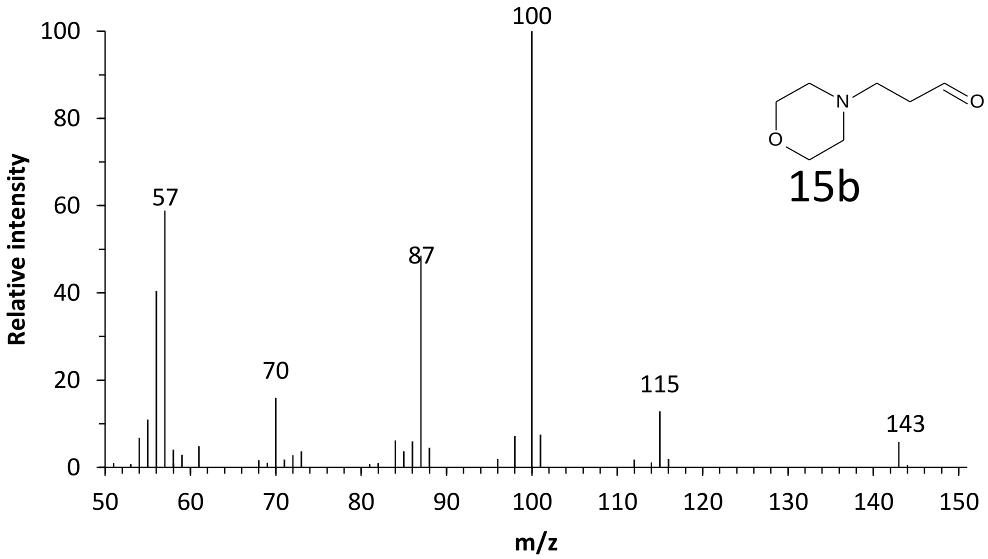

Relative intensity
m/z
57
70
87
100
115
143
N
O
O
15b

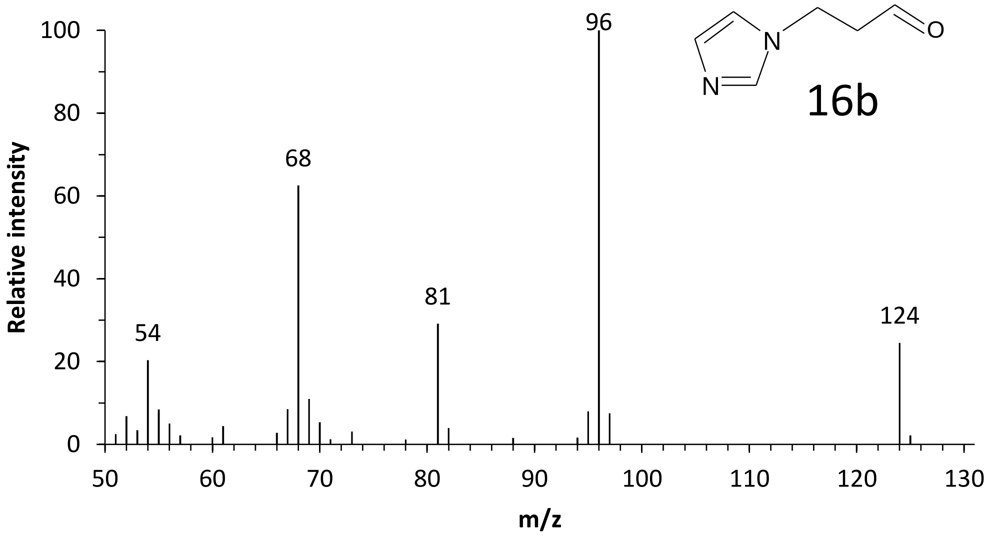

16b
N
N
O
96
68
81
54
124
100
80
60
40
20
0
Relative intensity
50
60
70
80
90
100
110
120
130
m/z

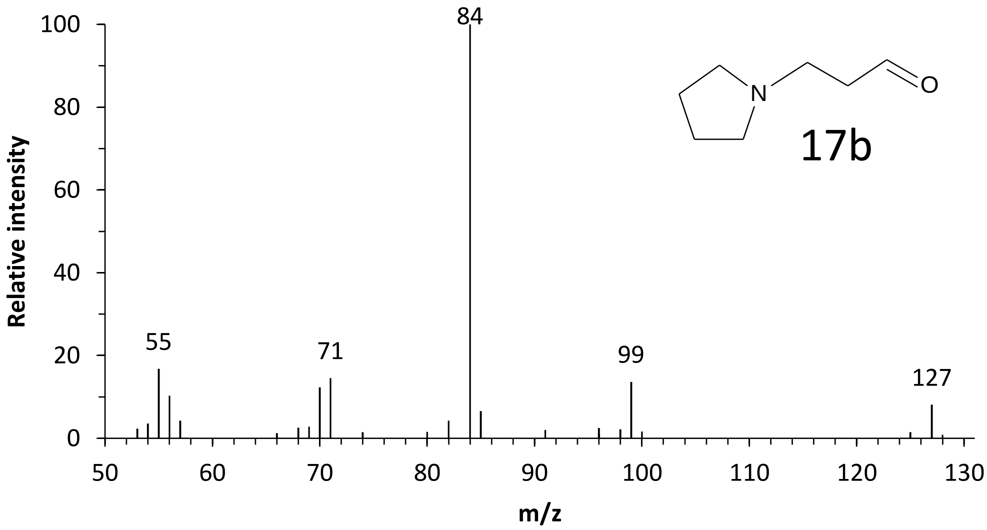

17b
N
O
84
55
71
99
127
100
80
60
40
20
0
Relative intensity
50
60
70
80
90
100
110
120
130
m/z

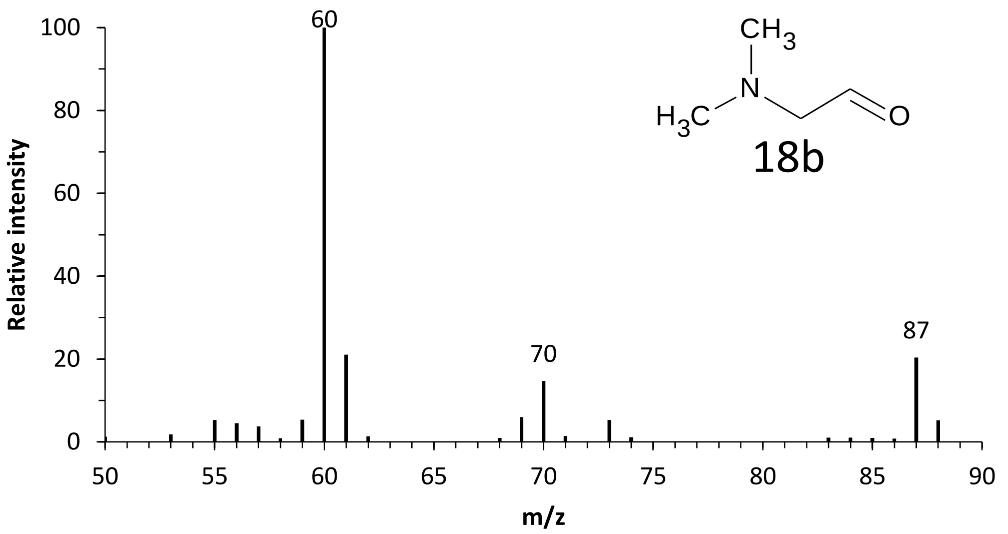
Relative intensity
m/z
100
80
60
40
20
0
50
55
60
65
70
75
80
85
90
60
70
87
CH3
N
H3C
O

18b

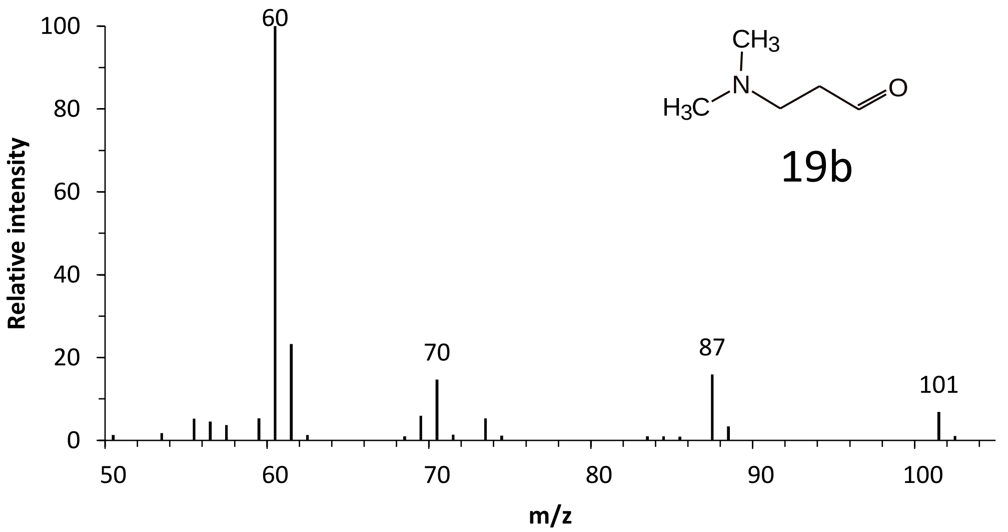
Relative intensity
m/z
100
80
60
40
20
0
50
60
70
80
90
100
60
70
87
101
CH3
N
H3C
O

19b

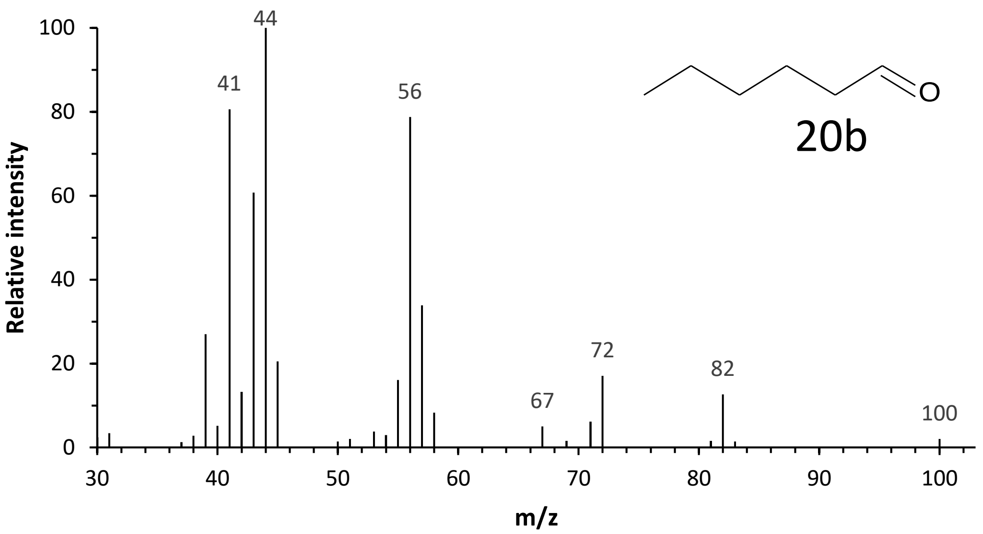

20b
O
Relative intensity
m/z
100
80
60
40
20
0
30
40
50
60
70
80
90
100
44
41
56
67
72
82
100

Figure S6: **Kinetic plots of LCAO catalyzed deamination of compounds 1a-8a, 10a-20a.**

All the kinetic parameters were obtained from the Michaelis and Menten plots. Initial rates of reaction were determined at the various substrate concentrations in the presence of 150 nM LCAO (20µg, 1 U), at 25°C by means of a coupled LCAO-peroxidase spectrophotometric assay. The formation of the colored product was followed at 515 nm for 1 min. Each point in the plot is the mean value of 3 replicates (SD values were always less than 5%). V_0 is expressed as µM min^{-1}.

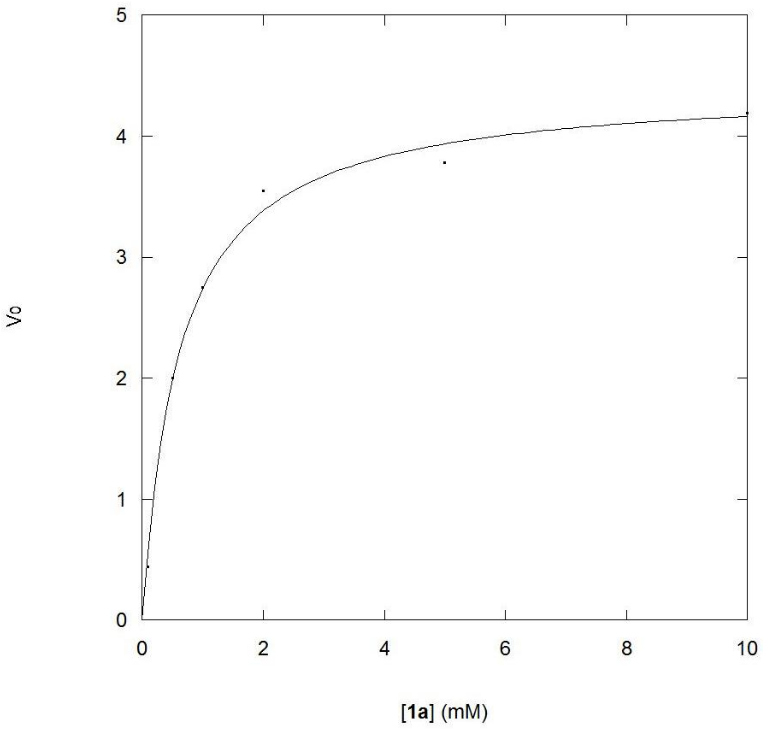

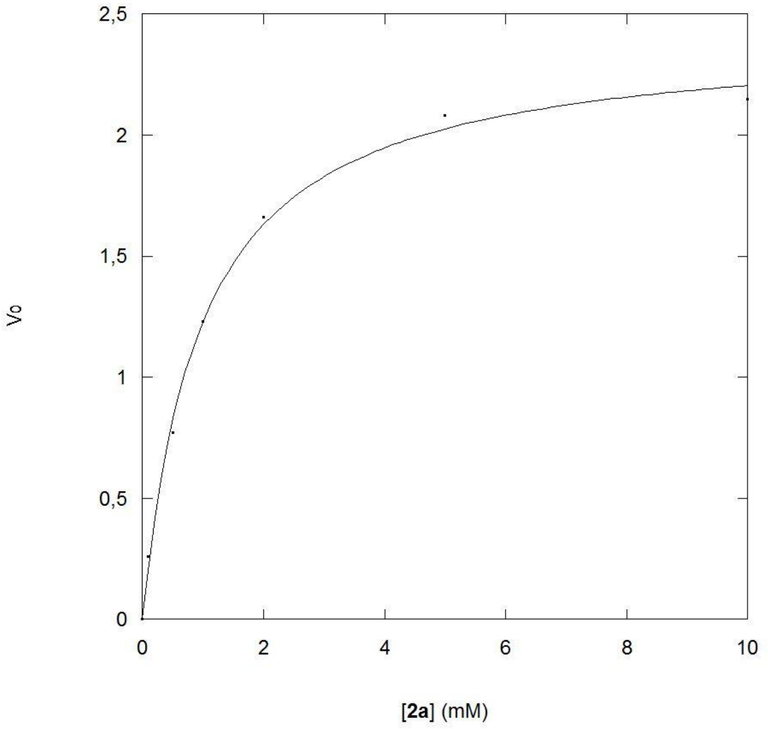
2,5
2
1,5
1
0,5
0
V0
0
2
4
6
8
10
[2a] (mM)

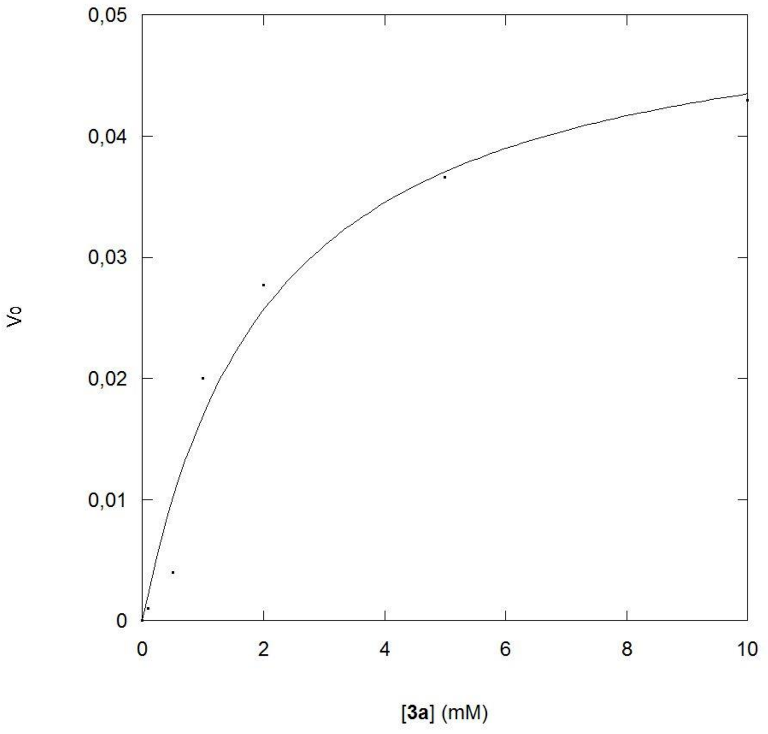
0,05
0,04
0,03
0,02
0,01
0
V0
0
2
4
6
8
10
[3a] (mM)

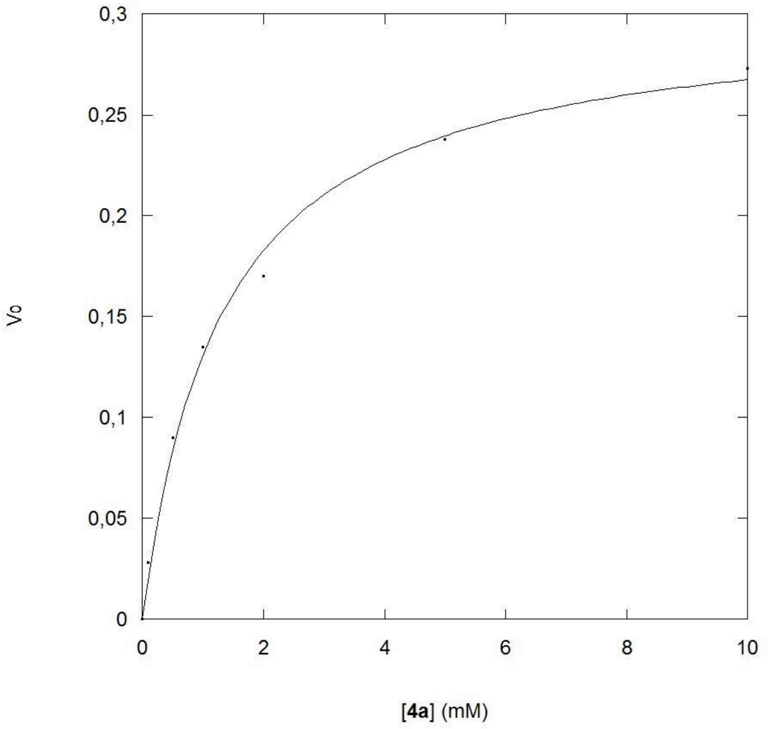
0,3
0,25
0,2
0,15
0,1
0,05
0
V0
0
2
4
6
8
10
[4a] (mM)

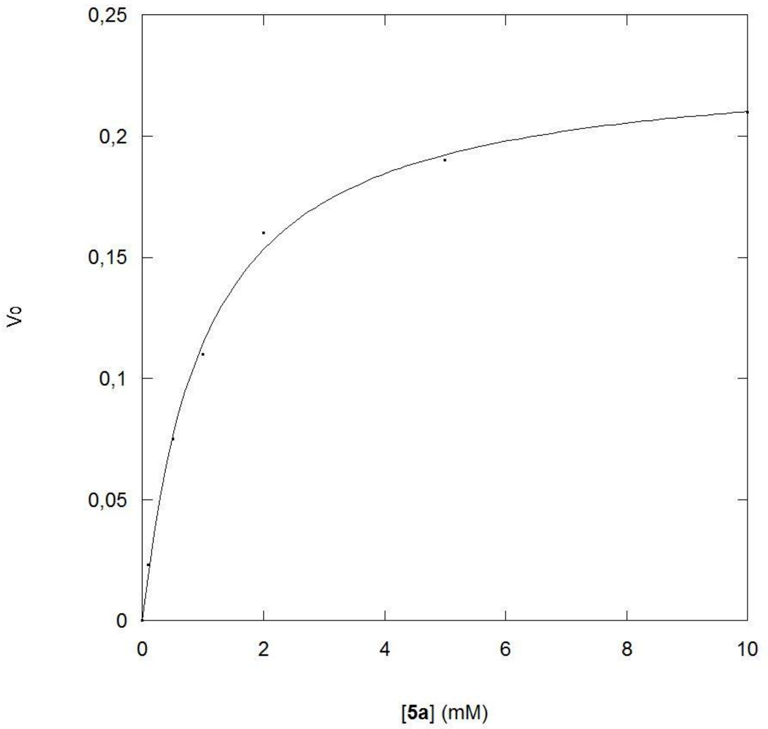
0,25
0,2
0,15
0,1
0,05
0
V0
0
2
4
6
8
10
[5a] (mM)

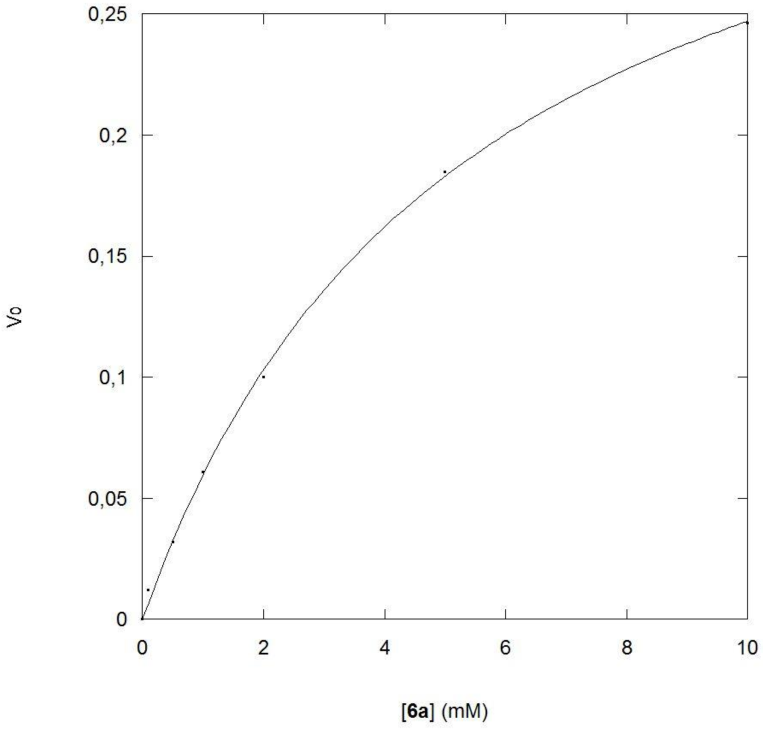
0,25
0,2
0,15
0,1
0,05
0
V0
0
2
4
6
8
10
[6a] (mM)

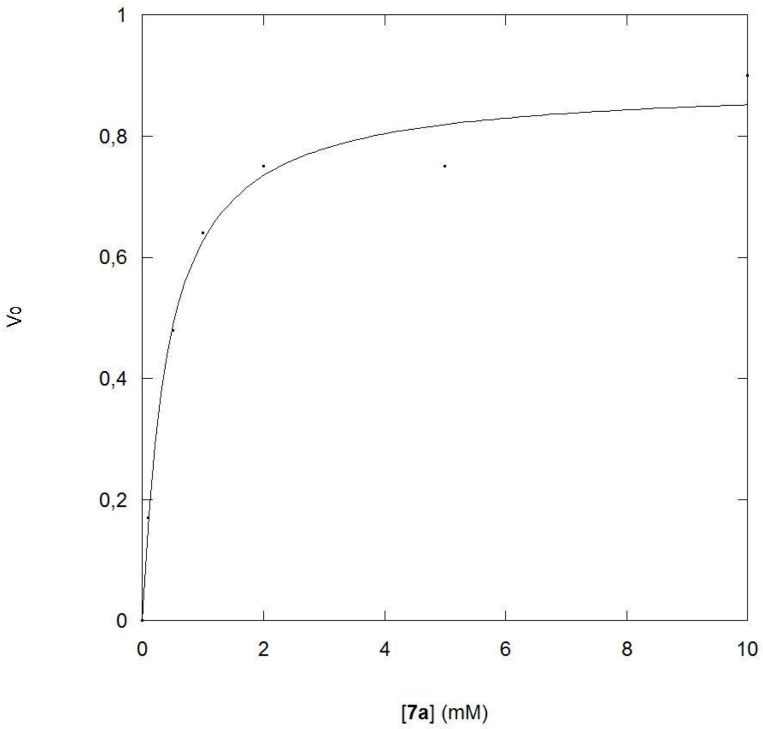
1
0,8
0,6
0,4
0,2
0
V0
0
2
4
6
8
10
[7a] (mM)

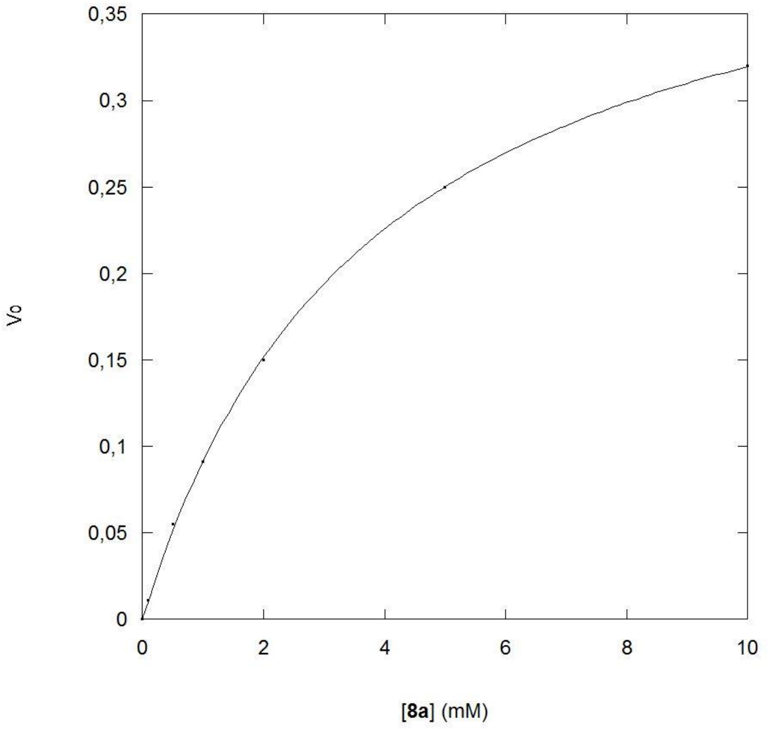
0,35
0,3
0,25
0,2
0,15
0,1
0,05
0
V0
0
2
4
6
8
10
[8a] (mM)

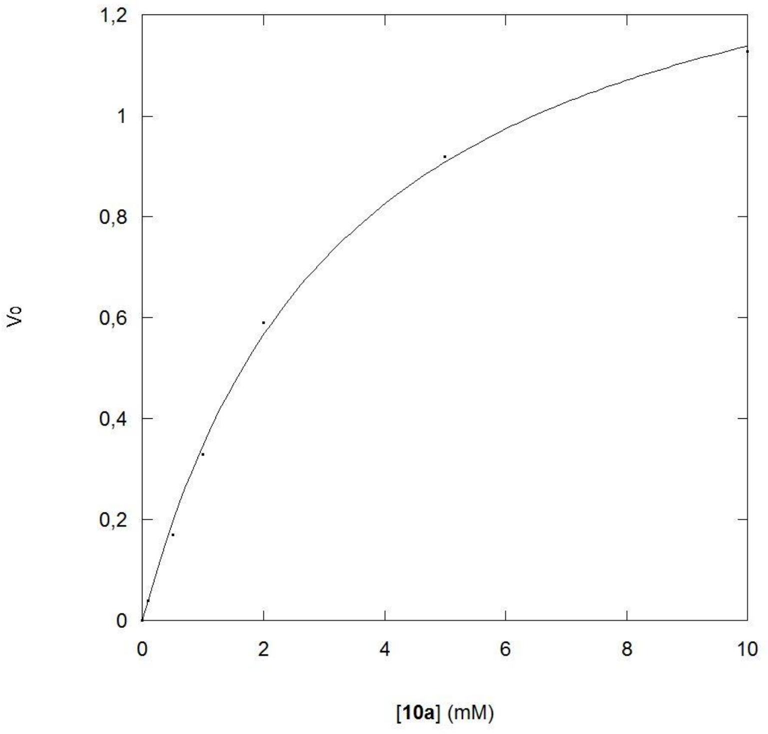
1,2
1
0,8
0,6
0,4
0,2
0
V0
0
2
4
6
8
10
[10a] (mM)

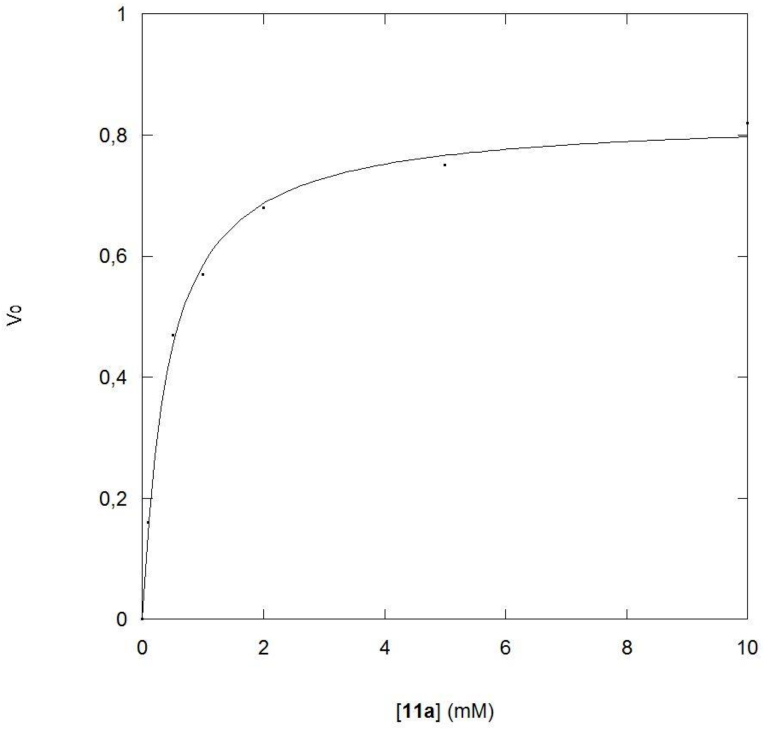
1
0,8
0,6
0,4
0,2
0
V0
0
2
4
6
8
10
[11a] (mM)

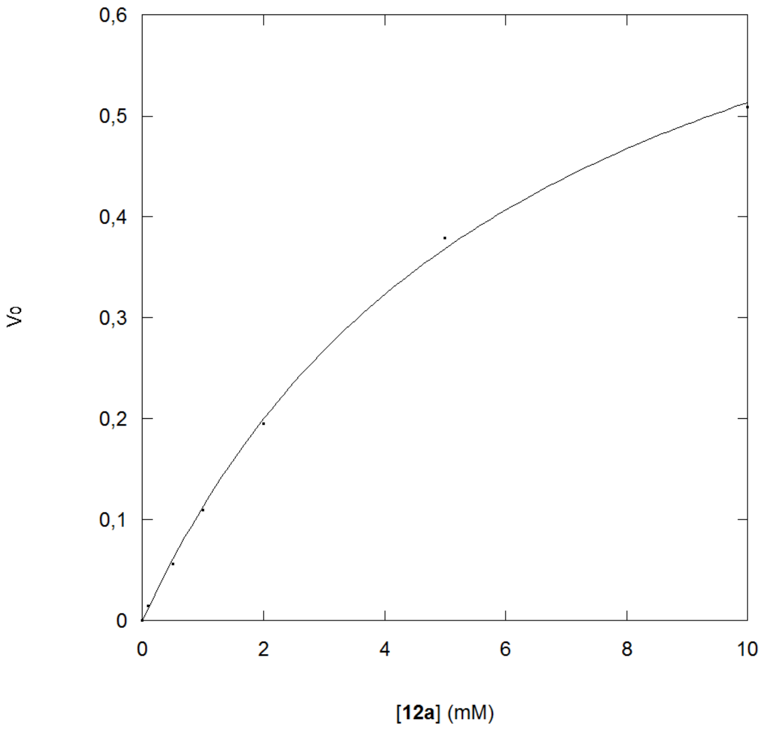
0,6
0,5
0,4
0,3
0,2
0,1
0
V0
0
2
4
6
8
10
[12a] (mM)

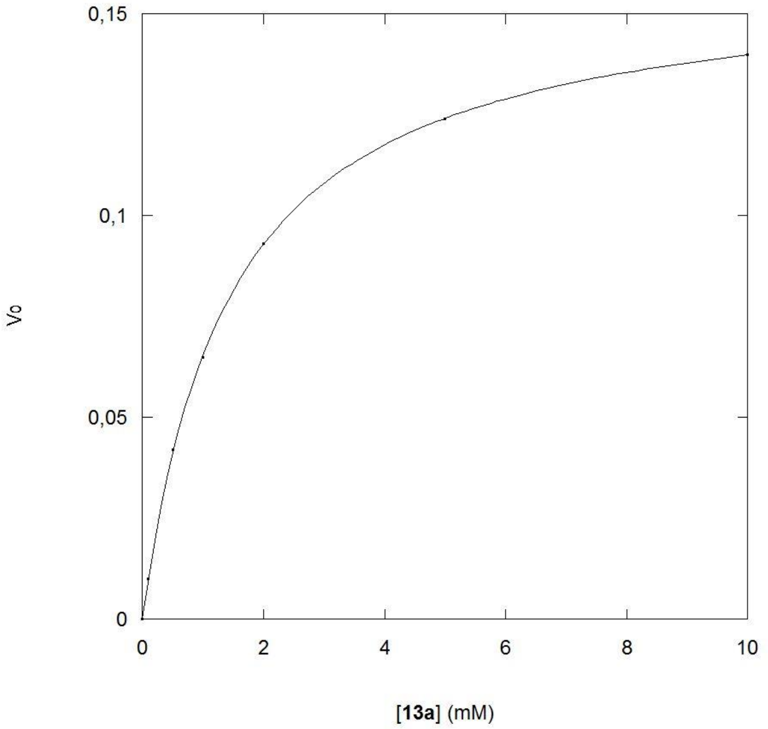
0,15
0,1
0,05
0
V0
0
2
4
6
8
10
[13a] (mM)

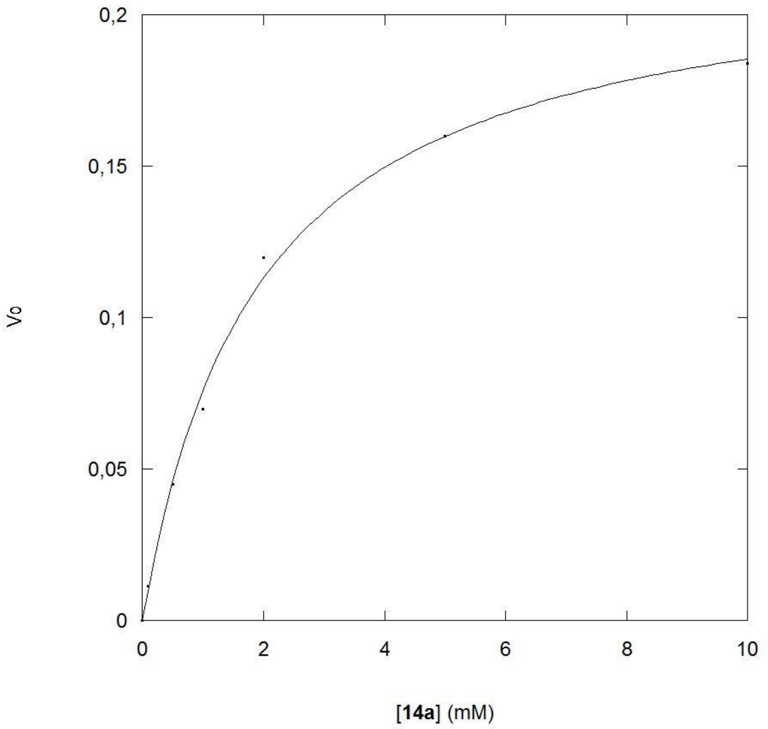
0,2
0,15
0,1
0,05
0
V0
0
2
4
6
8
10
[14a] (mM)

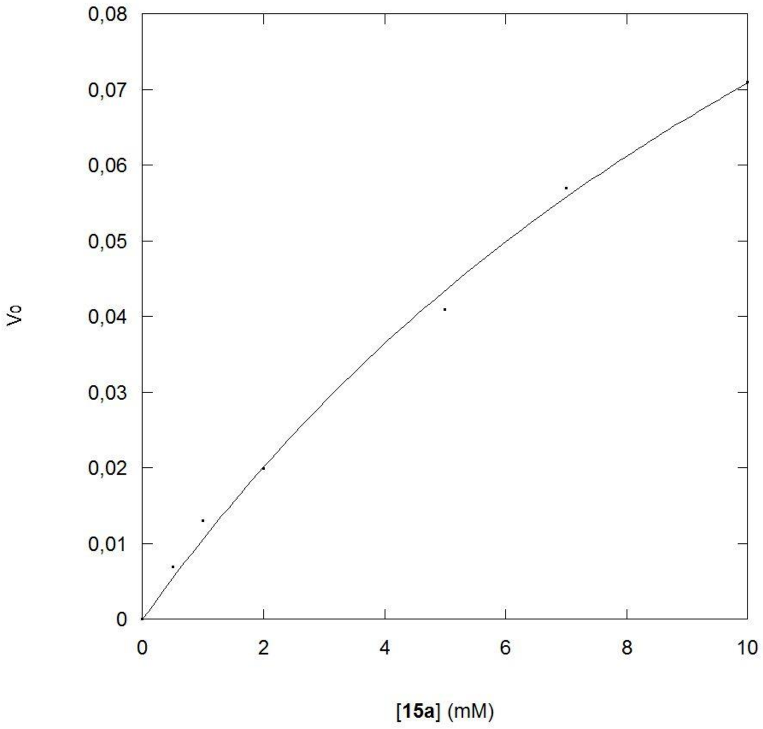

0,08
0,07
0,06
0,05
0,04
0,03
0,02
0,01
0
V0
0
2
4
6
8
10
[15a] (mM)

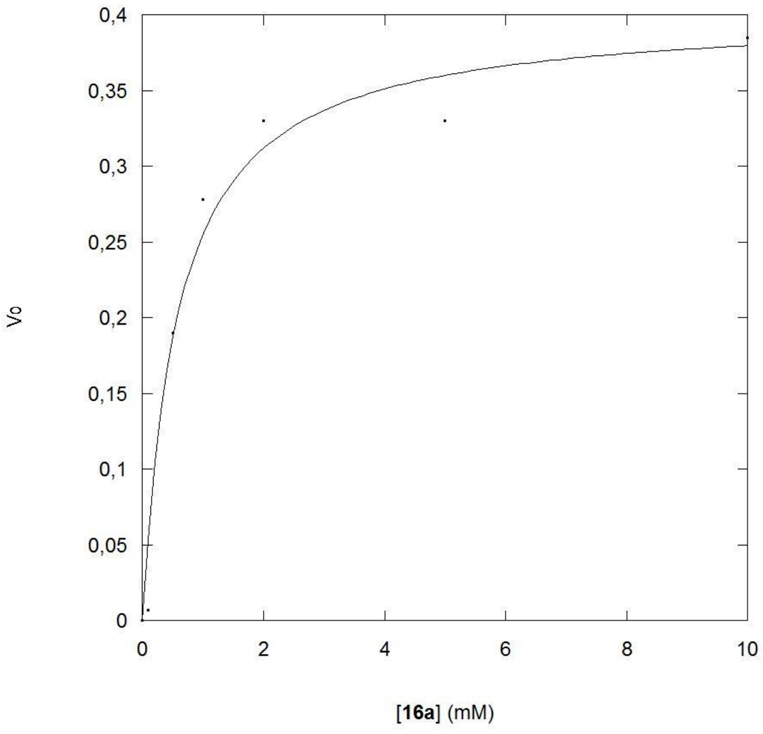

0,4
0,35
0,3
0,25
0,2
0,15
0,1
0,05
0
V0
0
2
4
6
8
10
[16a] (mM)

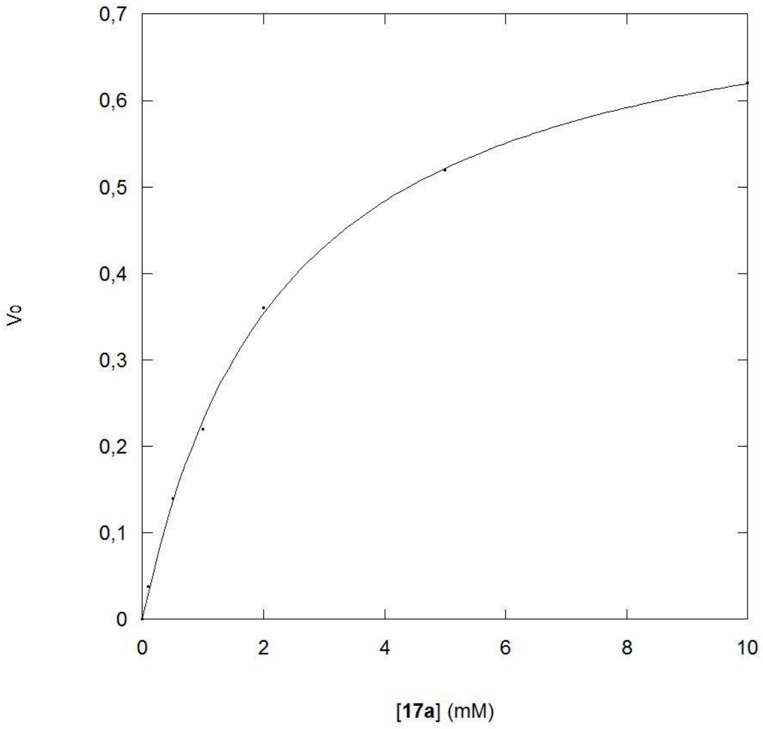

0,7
0,6
0,5
0,4
0,3
0,2
0,1
0
V0
0
2
4
6
8
10
[17a] (mM)

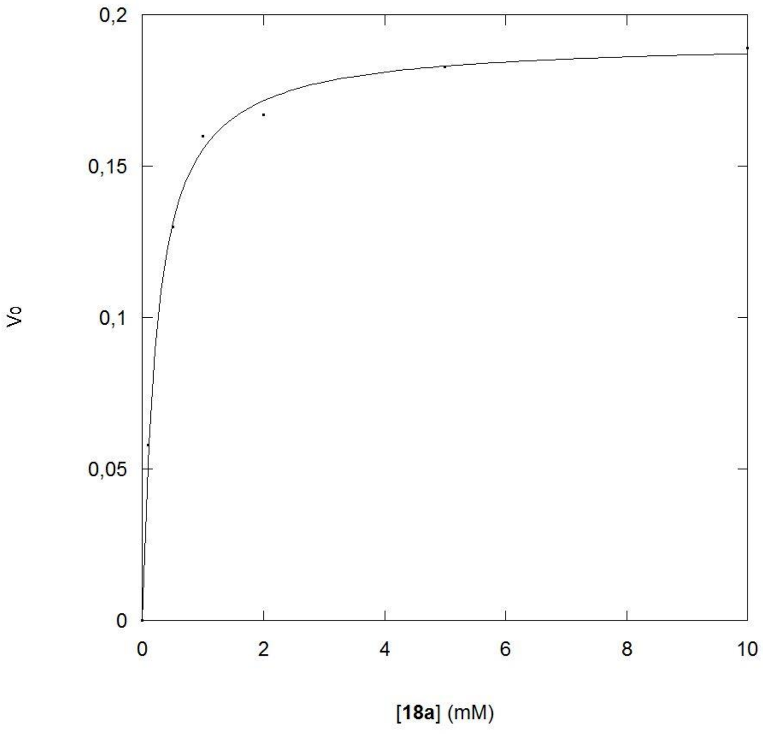

0,2
0,15
0,1
0,05
0
V0
0
2
4
6
8
10
[18a] (mM)

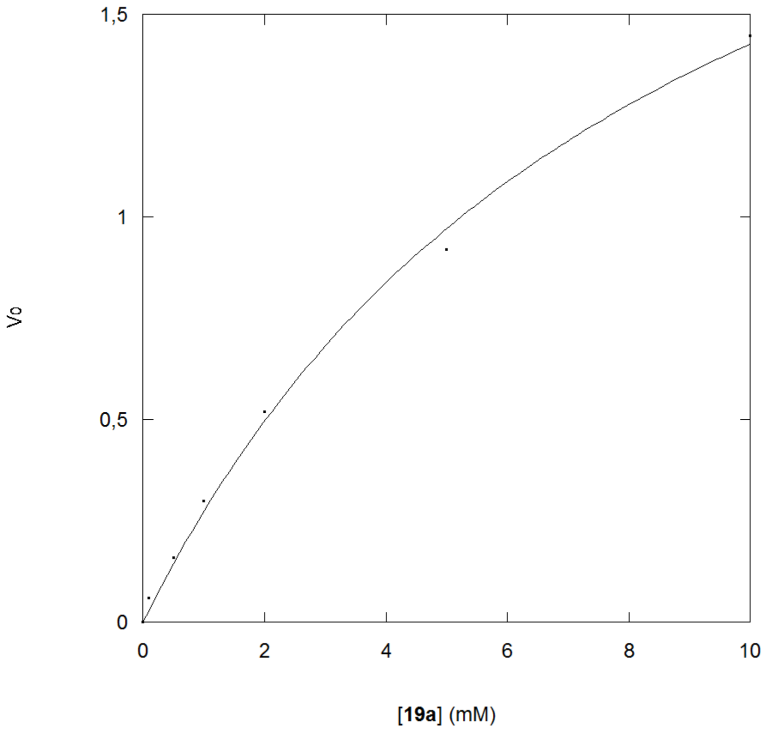

1,5
1
0,5
0
Vo
0
2
4
6
8
10
[19a] (mM)

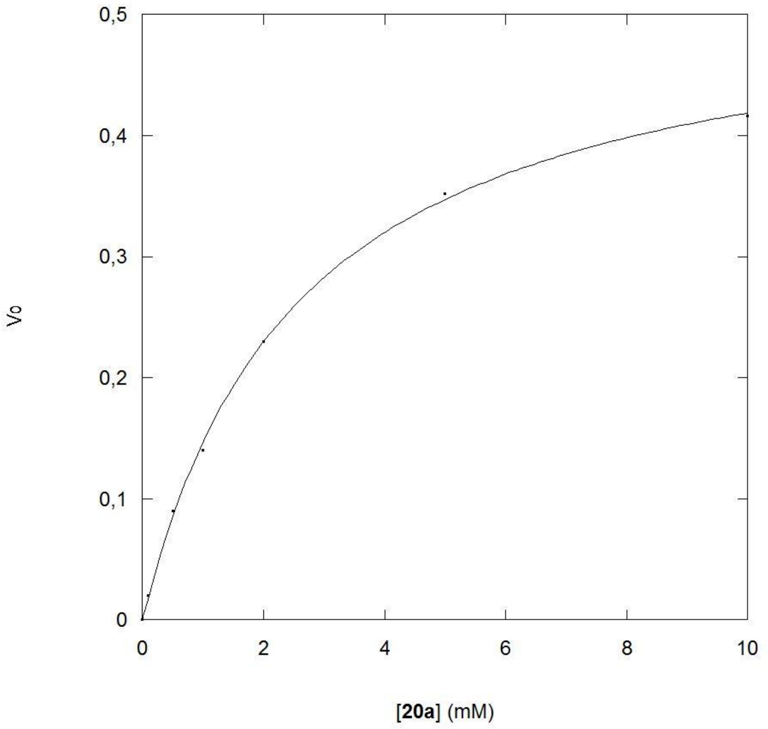

0,5
0,4
0,3
0,2
0,1
0
Vo
0
2
4
6
8
10
[20a] (mM)

Figure S7: Enzymatic synthesis of phenylacetaldehyde (compound 2b): HPLC and GC/MS analyses

As a proof of concept, a scale-up of the synthesis of compound **2b** was performed. Compound **2a** (78.5 mg, 5 mM, 100 mL final volume) in the presence of 150 U of LCAO was completely converted in compound **2b** in 1 hr. 100 U of LCAO were added to start the reaction and further 50 U were added to the reaction mix after 30 min. The reaction was followed by HPLC analyzing aliquots of the reaction mix with a Agilent Infinity 1260 HPLC apparatus equipped with UV detector. The separation was carried out as reported in "Materials and Methods" (section 2.4, main text).

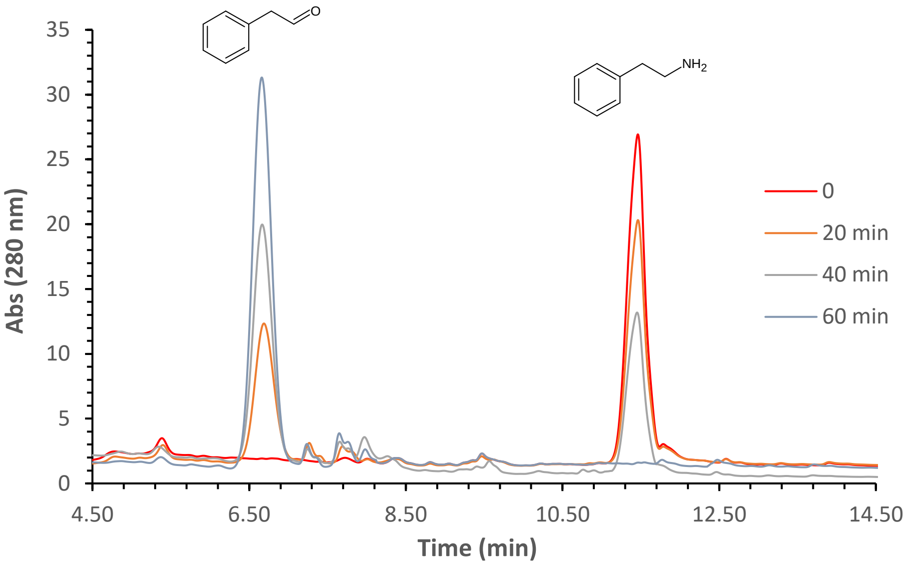

Once **2a** was completely converted in **2b**, the reaction mix was extracted twice with 1 vol. diethyl ether. The organic phase was dried and the resulting colorless liquid was weighted to 42.96 mg corresponding to a yield of isolated product (**2b**) of 71.6%. Isolated **2b** was analyzed by GC/MS to test its purity.

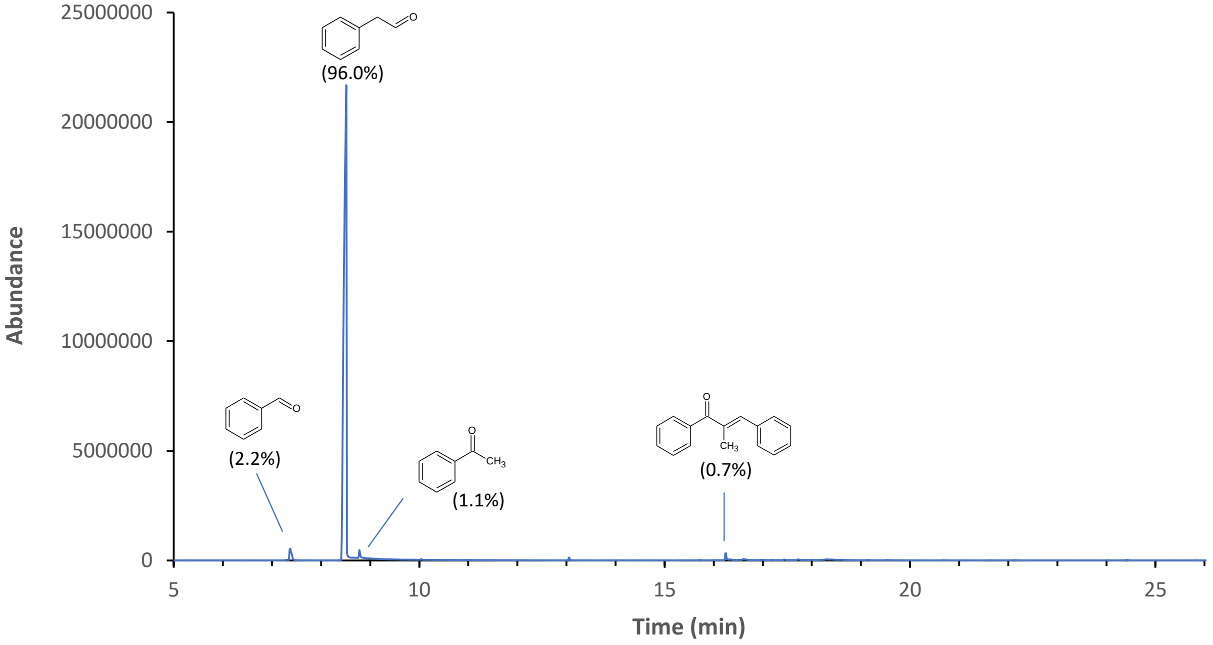

GC-MS chromatogram shows a purity of 96%. All the peaks were identified by comparison with those present in the NIST2017 database. The compound eluting at 16.27 min corresponds to an aldol dimerization product and may form during the extraction/concentration steps. Compounds eluting at 7.42 min and 8.45 min probably originate from LCAO-catalyzed transformation of amines present as impurities in **2a**.

Table S1: **Conversion of the primary amines 1a-8a, 10a-20a to the corresponding aldehydes 1b-8b, 10b-20b.**

n.	reaction product	Conversion (%)			
		0.5 h	1 h	2 h	3 h
**1b**	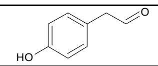	87	99		
**2b**	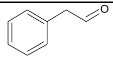	63	98		
**3b**	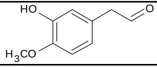	13	22	54	95
**4b**	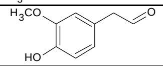	23	38	62	97
**5b**	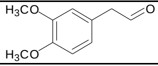	17	33	72	96
**6b**	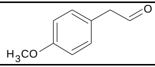	34	47	75	98
**7b**	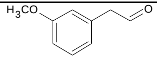	41	56	83	99
**8b**	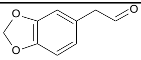	29	41	69	95
**10b**	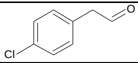	98			
**11b**	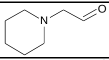	67	98		
**12b**	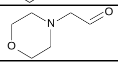	56	87	99	
**13b**	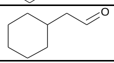	25	54	78	98
**14b**	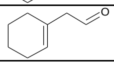	27	60	74	97
**15b**	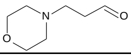	60	79	96	
**16b**	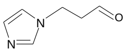	45	82	96	
**17b**	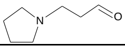	43	76	97	
**18b**	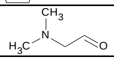	27	47	66	98
**19b**	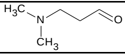	99			
**20b**	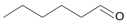	68	99		

0 100

Table S2: **Retention times of compounds 1a-8a, 10a-20a, 1b-8b, 10b-20b.**

**compound**	**HPLC (280 nm) retention time**	**GC/MS (ECF derivative)**	**compound**	**GC/MS**
**1a**	4.05	/	**1b**	12.23
**2a**	11.44	/	**2b**	8.47
**3a**	4.57	/	**3b**	11.55
**4a**	4.42	/	**4b**	11.48
**5a**	5.13	/	**5b**	11.82
**6a**	5.43	/	**6b**	9.27
**7a**	5.46	/	**7b**	9.07
**8a**	5.35	/	**8b**	10.34
**10a**	6.22	/	**10b**	8.61
**11a**	/	19.89	**11b**	7.67
**12a**	/	15.07	**12b**	7.64
**13a**	/	11.02	**13b**	7.00
**14a**	/	11.28	**14b**	5.67
**15a**	/	16.33	**15b**	7.31
**16a**	6.23	/	**16b**	9.36
**17a**	/	10.87	**17b**	11.62
**18a**	/	9.64	**18b**	4.27
**19a**	/	10.11	**19b**	4.30
**20a**	/	9.34	**20b**	4.98
